# Aggregation of Human Recombinant Monoclonal Antibodies Influences the Capacity of Dendritic Cells to Stimulate Adaptive T-Cell Responses *In Vitro*

**DOI:** 10.1371/journal.pone.0086322

**Published:** 2014-01-21

**Authors:** Verena Rombach-Riegraf, Anette C. Karle, Babette Wolf, Laetitia Sordé, Stephan Koepke, Sascha Gottlieb, Jennifer Krieg, Marie-Claude Djidja, Aida Baban, Sebastian Spindeldreher, Atanas V. Koulov, Andrea Kiessling

**Affiliations:** 1 Novartis Pharma AG, Technical R&D, Biologics Process R&D, Late Phase Analytical & Pharmaceutical Development, Werk Klybeck, Basel, Switzerland; 2 Novartis Pharma AG, Integrated Biologics Profiling Unit, Immunogenicity Risk Assessment, Werk Klybeck, Basel, Switzerland; 3 Novartis Pharma AG, Pre-clinical Safety, Biologics Safety and Disposition, Experimental Pathology, Immunosafety, Werk Klybeck, Basel, Switzerland; 4 Novartis Pharma AG, Pre-clinical Safety Biologics Safety and Disposition, Bioanalytics, Werk Klybeck, Basel, Switzerland; University of Pittsburgh, United States of America

## Abstract

Subvisible proteinaceous particles which are present in all therapeutic protein formulations are in the focus of intense discussions between health authorities, academics and biopharmaceutical companies in the context of concerns that such particles could promote unwanted immunogenicity via anti-drug antibody formation. In order to provide further understanding of the subject, this study closely examines the specific biological effects proteinaceous particles may exert on dendritic cells (DCs) as the most efficient antigen-presenting cell population crucial for the initiation of the adaptive immune response. Two different model IgG antibodies were subjected to three different types of exaggerated physical stress to generate subvisible particles in far greater concentrations than the ones typical for the currently marketed biotherapeutical antibodies. The aggregated samples were used in *in vitro* biological assays in order to interrogate the early DC-driven events that initiate CD4 T-cell dependent humoral adaptive immune responses – peptide presentation capacity and co-stimulatory activity of DCs. Most importantly, antigen presentation was addressed with a unique approach called MHC-associated Peptide Proteomics (MAPPs), which allows for identifying the sequences of HLA-DR associated peptides directly from human dendritic cells.

The experiments demonstrated that highly aggregated solutions of two model mAbs generated under controlled conditions can induce activation of human monocyte-derived DCs as indicated by upregulation of typical maturation markers including co-stimulatory molecules necessary for CD4 T-cell activation. Additional data suggest that highly aggregated proteins could induce *in vitro* T-cell responses. Intriguingly, strong aggregation-mediated changes in the pattern and quantity of antigen-derived HLA-DR associated peptides presented on DCs were observed, indicating a change in protein processing and presentation. Increasing the amounts of subvisible proteinaceous particles correlated very well with the pronounced increase in the peptide number and clusters presented in the context of class II HLA-DR molecules, suggesting a major involvement of a mass-action mechanism of altering the presentation.

## Introduction

Protein aggregation is the process of non-specific physical assembly of two or more protein molecules, which is usually the consequence of a certain level of unfolding of individual molecules and is generally driven by the hydrophobic effect. Protein aggregation is a ubiquitous phenomenon that takes place both, *in vivo* and *in vitro*. Both aspects being the focus of intense studies in the context of protein turnover [1], misfolding disease [2], [3] and adverse effects of protein aggregates in biotechnology products [4]. Due to the largely non-specific mechanism of protein aggregation, this process usually results in a very heterogeneous mixture of molecular products that vary structurally and span several orders of magnitude in size [5], ranging from nanometers (oligomeric species) to hundreds of micrometers (visible particles containing thousands of individual molecules). Most recently, subvisible proteinaceous particles which are inevitably present in protein solutions, have become the focus of intense discussions between health authorities, academics and biopharmaceutical companies with regard to the potential biological consequences for the innate and adaptive immune system [4], [6], [7]. The main question is how the human immune system responds to subvisible aggregate particles in human recombinant proteins, however, to this date, attempts to address this question in controlled laboratory experiments have remained very limited, and do not provide insights into the cellular mechanisms of potential aggregate induced immune reactions. The involvement of several different mechanisms in the potential influence of aggregates on the immune system has been discussed in the literature. For example, it has been speculated that due to partial unfolding microscopic aggregate particles may present a large number of non-native structures on their surface. Furthermore, due to the large number of molecules present in micrometer-sized particles, these non-native structures may be displayed in a semi-regular and repeated fashion. Such non-native surfaces could act as B-cell epitopes, causing direct B-cell activation in the absence of T-cell help by receptor clustering, ultimately leading to immunogenicity in terms of anti-drug antibody (ADA) formation [8].

In addition or as an alternative to direct B-cell activation, an increase in the uptake of the particulate material and presentation of the derived peptides by DCs as most important population of professional antigen presenting cells (APCs) could pose a possible trigger for specific CD4 T-cell responses [9]. A very recent study tested highly aggregated antibody therapeutics in a peripheral blood mononuclear cell (PBMC) *in vitro* system with a different focus [10]. This work mainly addressed the role of aggregates in the induction of innate immune responses and the potential links between innate and adaptive immune responses, which is largely complementary to our work studying the aggregate impact on the central mechanisms to induce adaptive immune responses. Results of Joubert at el. [10] showed a correlation between particle counts and an increased production of several innate cytokines compared to the unstressed materials. Toll-like receptors (TLR), Fc gamma receptors (FcγR) and complement factors were identified as mechanistic contributors to the response and, furthermore, increased adaptive immune responses to the aggregated material were detected by CD4 T-cell proliferation and activation. These findings already indicated a major involvement of HLA-restricted antigen presentation as an early mechanism that may lead to ADA responses and strongly support the notion that this process is T-cell dependent, but the exact mechanisms of CD4 T-cell stimulation by DCs were not investigated in this study.

The induction of a CD4 T-cell dependent immune response requires the activation and maturation of DCs by pathogen-associated molecular patterns (PAMPs), by danger-associated molecular patterns (DAMPs) [11] or by ligation of aggregated antibodies or immune complexes to activating receptors on DCs (e.g. complement receptors, FcγR) [12]. Immature DCs effectively take up and process proteins while mature DCs are very efficient in presenting protein-derived peptides to CD4 T-cells in the context of HLA class II [13]–[15]. In this regard, the genotype of the highly polymorphic HLA class II molecules determines the sequence of the peptides that can be bound. In addition, DCs express co-stimulatory molecules required for activation of antigen specific T-cells. T-cells activated by DCs can induce activation of B cells expressing B cell receptors specific for the very same antigen, finally leading to the secretion of antigen specific antibodies. The combination of all these factors including the maturation state of DCs, their HLA class II dependent pattern of presented peptides, the presence of specific CD4 T-cells and of B cell epitopes on the antigen are pieces of a puzzle which can lead to an antibody response to the antigen. Because of its impact on biotechnology and healthcare, the purported propensity of subvisible proteinaceous particles to elicit an immune response requires further studies, especially regarding the mechanisms by which aggregates could potentially shift the balance of the adaptive immune system towards immune recognition and reaction. While the gaps in the current knowledge are founded in the limited experimental models available to scientists today, it is important to continue addressing these questions by designing novel experimental approaches [16] [11].

This study is aimed at decoding the biological effects that aggregated fully human monoclonal antibodies (mAbs) may exert onto DCs, key players at stimulating the human adaptive immune system and at identifying the initiating cellular and molecular events that may play a role for induction of adaptive CD4 T-cell-mediated immune responses required for the formation of ADA. These early events involve activation of DCs by upregulation of co-stimulatory molecules, increased or changed presentation of aggregated therapeutic protein-derived peptides in the context of HLA-DR, the major subclass of HLA class II molecules, that together are required to finally effectively induce CD4 T cell responses. Two different humanized model IgG antibodies were subjected to different types of physical stress resulting in aggregation and formation of large numbers of subvisible particles. The physicochemical properties of these aggregate samples were characterized in detail using an array of analytical methods: Microflow Imaging (MFI), Size Exclusion Chromatography (SEC), Dynamic Light Scattering (DLS), Capillary Electrophoresis Sodium Dodecyl Sulfate (CE-SDS) and LC/MS peptide mapping. These well characterized aggregated samples were used in *in vitro* assays to investigate DC maturation (DC activation assay), antigen presentation (MHC associated Peptide Proteomics (MAPPs) assay) [17] and CD4 T-cell activation (T-cell assay).

The experimental results obtained from the *in vitro* assays show that aggregates of both model mAbs can induce activation of human monocyte-derived DCs as indicated by upregulation of typical maturation markers including co-stimulatory molecules crucial for CD4 T-cell activation whereby high levels of subvisible proteinaceous particles were associated with an elevated overall maturation.

Most importantly, strong aggregation-mediated changes in the pattern of model mAb-derived HLA-DR associated peptides on DCs were observed as determined via LC-MS. To our understanding, this is the first study analyzing the consequences of aggregation on presentation of HLA-DR associated peptides. Increasing amounts of subvisible proteinaceous particles correlated very well with the number of different presented peptides as well as with an increase in the number of nested peptide clusters presented in the context of HLA-DR, which suggests a mechanism leading to alteration of a CD4-driven immune response to highly aggregated proteins as confirmed in a small donor panel.

## Results

### Generation and characterization of protein aggregates

In the scope of this study, two different model mAbs (both of IgG1-subclass) targeting soluble proteins in plasma were used to generate aggregated preparations. Three different types of physical stress were applied to the model mAbs including freeze/thaw stress (FT), shear stress (S) and heat/shaking stress (HS) (see Materials and Methods for further details). The first two stress methods were chosen because of their general relevance to the stress that biopharmaceutical products may experience, although the intensities used were highly exaggerated in order to maximize the effects. Given the mechanistic nature of these studies a third set of conditions (HS) was included using an additional, completely artificial stress method. In order to assess potential dose-dependent effects of the subvisible particles in the aggregate preparations in the biological *in vitro* assays, each stress condition was applied at two different levels with increasing stress intensity, resulting in unstressed (un), stress level 1 (sl1) and stress level 2 (sl2) material (see Materials and Methods section for detailed description).

The aggregate preparations produced in such manner were quantified and characterized at the same time as testing in the *in vitro* assays. For characterization of the different aggregate samples, SEC, DLS and MFI were performed to cover the entire size-range from an antibody monomer to visible particles. In addition, all samples were characterized by peptide mapping and CE-SDS under reduced and non-reduced conditions in order to confirm the absence of chemical modifications of the primary protein structure. Both techniques showed highly similar chromatograms and electropherograms in all samples indicating that the stress did not induce any detectable chemical modification on the material (Figure S1, Figure S2, Figure S3 and Figure S4). In order to generate control samples devoid of any microscopic particles, the unstressed samples were filtered using a 0.22 µm filter shortly before incubation with cells.

**Figure 1:**
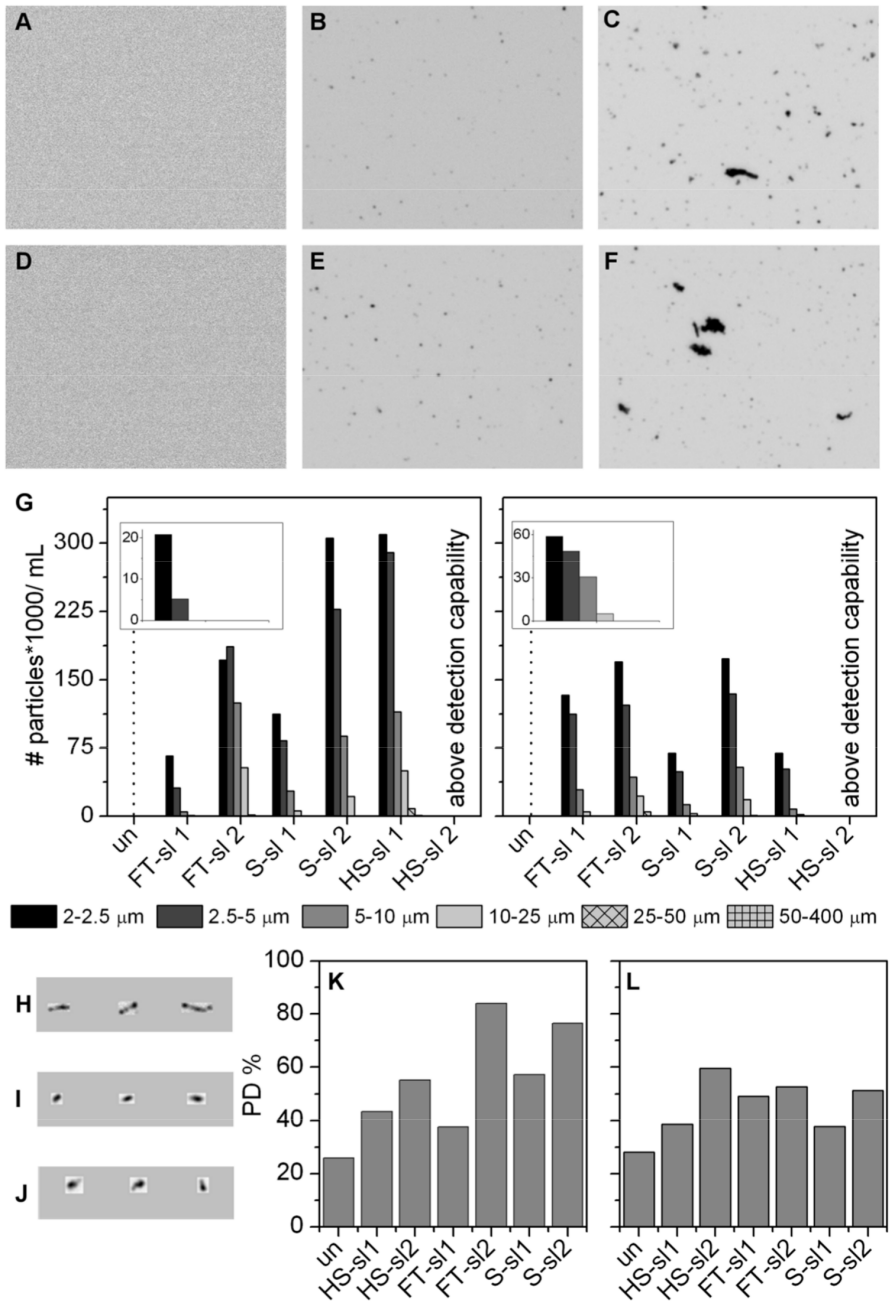
Physicochemical characterization of stressed mAb materials. Representative MFI screenshots after freeze/thaw (FT) stress of mAb1 (A) unstressed, un; (B) stress level 1, sl1; (C) stress level 2, sl2 and mAb2 un, (E) sl1 and (F) sl2. (G) Particle Size distribution obtained by MFI of mAb1 (left) and mAb2 (right). For visualization the size binning 2–2.5, 2.5–5, 5–10 10–25, 25–50 and 50–400 µm was used. Representative images of individual particles formed by (H) heat/shake, HS; (I) freeze/thaw, FT and (J) shear stress, S. Polydispersity in % (PD%) revealed by DLS for (K) mAb1 and (L) mAb2.

As depicted in Figure 1, the unstressed samples contain nearly no microscopic particles (detected by MFI) and very few nano-particles (as indicated by very low polydispersity by DLS). In contrast to the unstressed material, the amount of aggregates in the stressed samples rose with increase in stress intensity as indicated by the higher PD% values determined by DLS and higher particle counts by MFI as well as the loss of the monomer peak observed in SEC analysis (Figure S5). Thus, the increase in aggregates was most pronounced for sl2 material for all three stress conditions (Figure 1). This trend was similar for both proteins, although the total numbers of aggregates between the two proteins differed. All stress conditions resulted in similar particle size distributions, with most particles in the size range of 2 to 5 µm showing a decay distribution towards particles in the size range of 50–400 µm. Although identical stress levels were applied to both mAb1 and mAb2, the mAb1 samples contained more particles (by MFI and DLS) than the corresponding mAb2 preparations, likely reflecting the intrinsically different propensities of these proteins to form aggregates. This difference in aggregation propensities was also reflected by the SEC chromatograms showing that all mAb2 samples contained soluble aggregates, whereas for mAb1 just the loss of monomeric species was measurable while the amount of detectable soluble aggregates decreased. The loss of detectable aggregates might be caused by physical filtration, dilution or adsorption to surfaces during the SEC measurement (Figure S5).

In addition to differences in the extent of aggregation between the two model proteins used, differences in the particle counts and aggregate levels were observed between different stress methods. For example, HS stress resulted in the most dramatic increase of total particle counts from sl1 to sl2. The particle counts in HS sl2 samples of both mAbs could not be reliably quantified because the counts were well above the saturation limit of the MFI instrument and these samples also contained large amounts of visible particles which were not detected in the samples produced using S and FT stress methods.

Differences were also observed in the morphology of the particles resulting from the different stress techniques as shown by MFI, a method which offers the possibility to capture digital photographs of all measured particles and analyze the characteristic shape of the particles generated by different stress methods. Interestingly, the different stress methods applied in this study resulted in the generation of aggregates of different morphology. More specifically, aggregates generated by FT stress or S stress showed a much more rounded, dense and compact morphology as compared to the HS stress induced particles, which tended to be elongated and filamentous (Figure 1).

### Analysis of model mAbs on a protein array for evaluation of cross-reactivity

To rule out responses in the two bioassays related to unspecific binding to the cells due to aggregation of the model mAbs, a cross-reactivity assay was performed in which all aggregate preparations were tested for cross-reactivity to a panel of more than 400 proteins on a protein-array. All preparations showed cross-reactivity against less than 2.2% of the proteins on the protein-array which is below the assay cut-off of 2.48% cross-reactive proteins (data not shown). Accordingly, aggregation did not increase cross-reactivity to the proteins in any of the tested aggregate preparations.

### Maturation of dendritic cells by aggregate preparations

The capacity of the different aggregate preparations of mAb1 and mAb2 to induce maturation of immature monocyte-derived DCs from healthy donors was assessed at final concentrations of 10, 70 and 200 µg/ml. The upregulation of the maturation marker CD83 and the CD4 T-cell co-stimulatory molecules CD80 and CD86 relative to the corresponding unstressed mAb preparations was determined by fluorescence activated cell sorting analysis (FACS) via evaluation of the mean fluorescence intensities. Different panels of twelve, six and eight donors were tested for the S, FT and HS studies, respectively. The positive control (a cytokine cocktail as specified in Material and Methods) resulted in an efficient upregulation of CD83, CD80 and CD86 in all tested donors when compared to the negative controls (formulation buffer added at the same volume as mAbs and medium only control). Unstressed mAb1 samples already showed a concentration-dependent increase of marker expression compared to DPBS with an effect in most donors that was pronounced for CD86, whereas unstressed mAb2 showed only weak DC maturation effects in only a few donors (data not shown).

**Figure 2:**
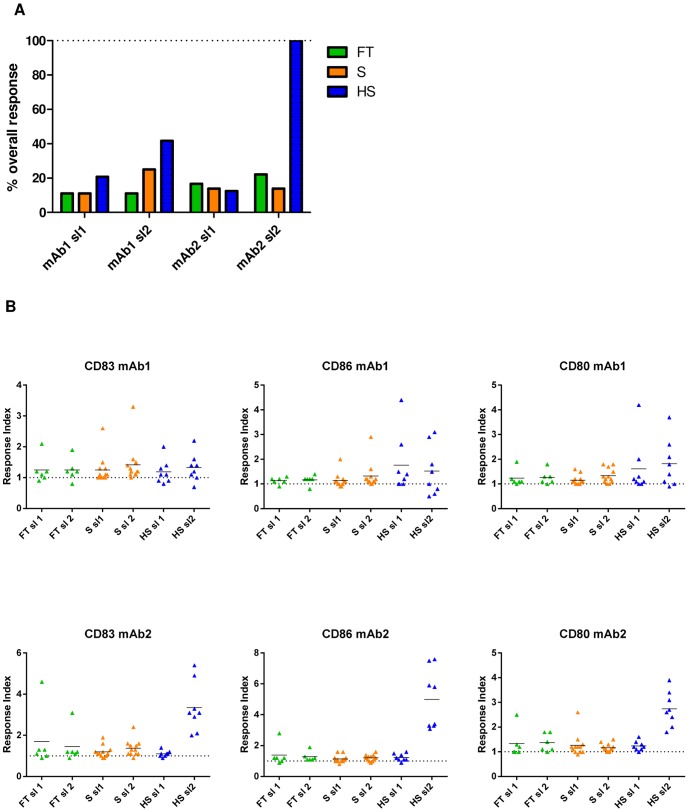
Induction of DC maturation by stressed mAb materials. (A) Percentage of responding donors determined as an at least 1.5-fold upregulation of the response index above unstressed mAb of the maturation markers CD83, CD80 and CD86. (B) Scatter plots of the response indices of individual donors for the measured maturation markers. The dotted line indicates the reference to unstressed mAb at 1.0. The horizontal line represents the average of the individual response indices. HS: aggregates generated by heat and shake stress, FT: aggregates generated by freeze and thaw stress, S: aggregates generated by shear stress, mAb1: monoclonal antibody 1, mAb2: monoclonal antibody 2, RI: Response index, sl1: stress level 1, sl2: stress level 2.

Figure 2B shows the individual upregulation of marker expression as fold increase above the unstressed mAb1 and mAb2 materials (named response index) for the respective concentration showing the highest response in each stress condition. For calculation of the percentage of responding donors averaged over all markers (% overall response, Figure 2A), weakly responding donors were neglected by setting a threshold of 1.5-fold upregulation. To illustrate the average magnitude of response for individual stress conditions and levels, the mean of the individual marker upregulation was calculated and expressed as average response index for each marker over all tested donors and displayed together with the significance levels when the mean fluorescence intensities for the stressed mAbs were compared with that of unstressed mAbs (paired t-test) (Table 1).

**Table 1:** Average response indices in the in vitro DC assay, mean increase of mAb-derived different peptides and clusters in the MAPPs assay and statistical significance for aggregate preparations.

Stress condition			S		FT		HS	
stress level			sl1	sl2	sl1	sl2	sl1	sl2
Average Response index: DC assay	mAb1	CD83	1.3	1.4	1.3	1.3	1.2	1.3
		CD86	1.1	1.3	1.1	1.2	1.8	1.5
		CD80	1.2	1.3	1.2	1.3	1.6	1.8
		all markers	1.2	1.4	1.2	1.2	1.5	1.6
	mAb2	CD83	1.2	1.4	1.7	1.5	1.1	3.4
		CD86	1.1	1.2	1.4	1.3	1.2	5.0
		CD80	1.3	1.2	1.3	1.4	1.2	2.7
		all markers	1.2	1.3	1.5	1.4	1.2	3.7
Significance level vs unstressed material: DC assay	mAb1	CD83	*	**	ns	ns	ns	ns
		CD86	ns	ns	*	ns	ns	ns
		CD80	*	**	*	*	*	*
		all markers	*	ns	ns	ns	ns	ns
	mAb2	CD83	ns	**	ns	ns	ns	***
		CD86	ns	*	ns	ns	*	***
		CD80	ns	**	ns	*	**	***
		all markers	ns	**	ns	*	**	***
Mean increase of different peptides/clusters per donor: MAPPs assay	mAb1	peptides	2.1	3.3	2.0	3.8	8.7	16.6
		clusters	1.5	2.0	1.6	2.4	5.1	8.7
	mAb2	peptides	1.1	1.8	1.5	2.3	1.2	5.0
		clusters	1.1	1.2	1.2	1.6	1.2	3.6
Significance level vs unstressed material: MAPPs assay	mAb1	peptides	*	**	***	***	**	***
		clusters	*	**	**	***	***	***
	mAb2	peptides	ns	***	***	***	*	***
		clusters	ns	ns	ns	**	ns	***
Significance level sl1 vs sl2 material: MAPPs assay	mAb1	peptides	**		***		***	
		clusters	*		**		***	
	mAb2	peptides	**		***		***	
		clusters	ns		**		***	

HS: aggregates generated by heat and shake stress, FT: aggregates generated by freeze and thaw stress, S: aggregates generated by shear stress, mAb1: monoclonal antibody 1, mAb2: monoclonal antibody 2, sl1: stress level 1, sl2: stress level 2, statistical test conditions: all given significance levels refer to the corresponding unstressed mAb preparation (for DC assay and MAPPs) or relate to a comparison between sl1 and sl2 (MAPPs assay only) after conducting a two-tailed paired t-test with 95% confidence; ns: not significant, >0.05;

* : 0.01–0.05;

** : 0.001–0.01;

*** : <0.001;

DC assay: all markers: for average response indices the mean of CD83, CD86 and CD80 was calculated; significance levels on all markers are based on average mean fluorescence intensity values of CD83, CD86 and CD80.

The HS condition at sl2 resulted in the strongest DC maturation for both mAb1 and mAb2. For HS sl1 material of mAb2 an increase of maturation marker expression was observed in most donors mostly below but close to the response threshold of 1.5-fold upregulation (Figure 2), still resulting in a significant difference in responses compared to unstressed material for CD80 (p = 0.0061) and CD86 (p = 0.0157) (Table 1). For sl2, mAb2 showed very strong maturation capacity by upregulation of all three markers in all 8 donors (100% overall response, Figure 2) and high individual response indices of 2.0 to 5.4 for CD83, 3.1 to 7.6 for CD86 and a 1.8 to 3.9 for CD80, respectively (Figure 2B). This maturation effect was highly significant for all markers (p<0.001) (Table 1). Similarly for mAb1, a difference between the both HS stress levels was seen, although less pronounced. Significant differences to unstressed material were only found for CD80 sl1 (p = 0.0474) and sl2 (0.0131). The overall response rates above the threshold of 1.5-fold upregulation for sl1 and sl2 of mAb1 were determined to be 21% and 42%, respectively (Figure 2A).

In most cases, the overall response rates above the threshold of 1.5 fold upregulation and the average response indices over all markers at the corresponding stress levels were lower for the FT and S studies when compared to the HS study. The response indices for individual donors for both mAbs were below 2-fold in the majority of tested donors for both FT and S stress levels, which is also reflected in the lower overall response rates (Figure 2).

In the case of S samples incubation with sl2 material of mAb1 resulted in a slightly higher overall marker upregulation and percent response when compared with sl2 material of mAb2 (Figure 2 and Table 1). This upregulation was significantly above the unstressed material for CD83 (p = 0.0051) and CD80 (p = 0.0015) for mAb1 and for all markers (0.001≤p≤0.05) for mAb2. For sl1 material, only mAb1 showed statistically significant upregulation for the maturation markers CD83 (p = 0.0454) and CD80 (p = 0.0356) (Table 1).

For the FT samples a slight upregulation of marker expression was found in most donors stimulated with mAb1 and mAb2 at both stress levels with similar tendencies (Figure 2 and Table 1).

When the DC maturation capacity was correlated with the total number of subvisible particles measured by MFI and tested for a monotonic relationship (Spearman's rank correlation coefficient), clear correlations were found for the total particle count over the full MFI detection range (2–400 µm) with rank correlation coefficients of 0.943 and 0.751 for mAb1 and mAb2, respectively. Consequently, increased particle counts are associated with higher levels of DC maturation.

### Aggregate processing (MHC associated Peptide Proteomics - MAPPs assay)

#### Increase of model antibody-derived different peptides

**Figure 3:**
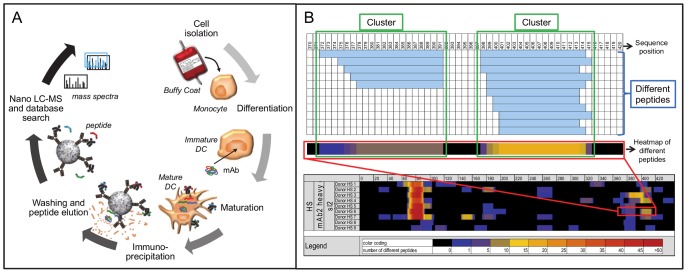
Illustration of MAPPs assay procedure and data output example. A: Illustration of MAPPs assay procedure. Monocytes are isolated from human buffy coats and differentiated to immature DCs in the presence of cytokines. Immature DCs are loaded with the model mAb and induced to maturation with lipopolysaccharide. After 24 hours, mature DCs are frozen. After lysis of mature DCs, HLA-DR:peptide complexes are isolated via immunoprecipitation using anti-HLA-DR coated beads. After several wash steps, peptides are eluted from HLA-DR complexes by pH shift and analysed by nano LC-MS with subsequent sequence identification via SEQUEST database search. B: MAPPs assay data output example. HLA-DR associated peptides can originate from different sequence regions of a protein and can occur in multiple length variants. Peptides in a sample with unique sequence are termed “different peptides”, highlighted with blue box. Nested sets of peptide length variants occurring within a sequence region sharing the same HLA-DR binding core are termed “clusters”, highlighted with green boxes. The number of different peptides per amino acid position can be summarized in a heatmap in which the cell colors are reflecting the number of different peptides, highlighted with red box. Different donors can differ in the pattern of presented peptides depending on binding propensities of their 2 HLA-DR alleles.

Utilizing the unique MAPPs assay, naturally presented HLA-DR associated peptides could be identified directly from human DCs (Figure 3). Immature monocyte-derived DCs from healthy human donors were loaded with different aggregate preparations of the model mAbs at a final concentration of 70 µg/ml in the presence of 1 mg/ml LPS to induce full DC maturation for most efficient antigen presentation. HLA-DR restricted peptides were isolated from mAb-loaded DC cultures and the sequence of the presented peptides was determined via liquid chromatography-mass spectrometry.

HLA-DR associated peptides in samples derived of DCs are highly diverse in terms of sequence lengths and originate from a variety of proteins which are naturally present in the endolysosomal compartment such as enzymes or HLA molecules, or from proteins that are taken up during macropinocytosis or receptor-mediated endocytosis. Thus, the samples contain a highly complex and diverse mixture of peptides with only a small portion of model mAb-derived peptides, which requires a high assay sensitivity. HLA-DR associated peptides can originate from different sequence regions of a protein and usually occur in multiple length variants. Peptides in a sample with unique sequence are hereinafter termed “different peptides”. Nested sets of peptide length variants occurring within a sequence region sharing the same HLA-DR binding core are hereinafter termed “clusters”.

In the three studies using S, FT and HS material, averages of 1574, 1484 and 1099 different peptides originating from a variety of proteins were identified per 5×10^6^ dendritic cells, demonstrating the high assay sensitivity. The variation in the number of different peptides between samples of the same donor was low, indicating good reproducibility of the method. The three studies using S, FT and HS material were performed on different donor sets not fully overlapping regarding their HLA-DRB1 haplotypes (Table S1).

**Figure 4:**
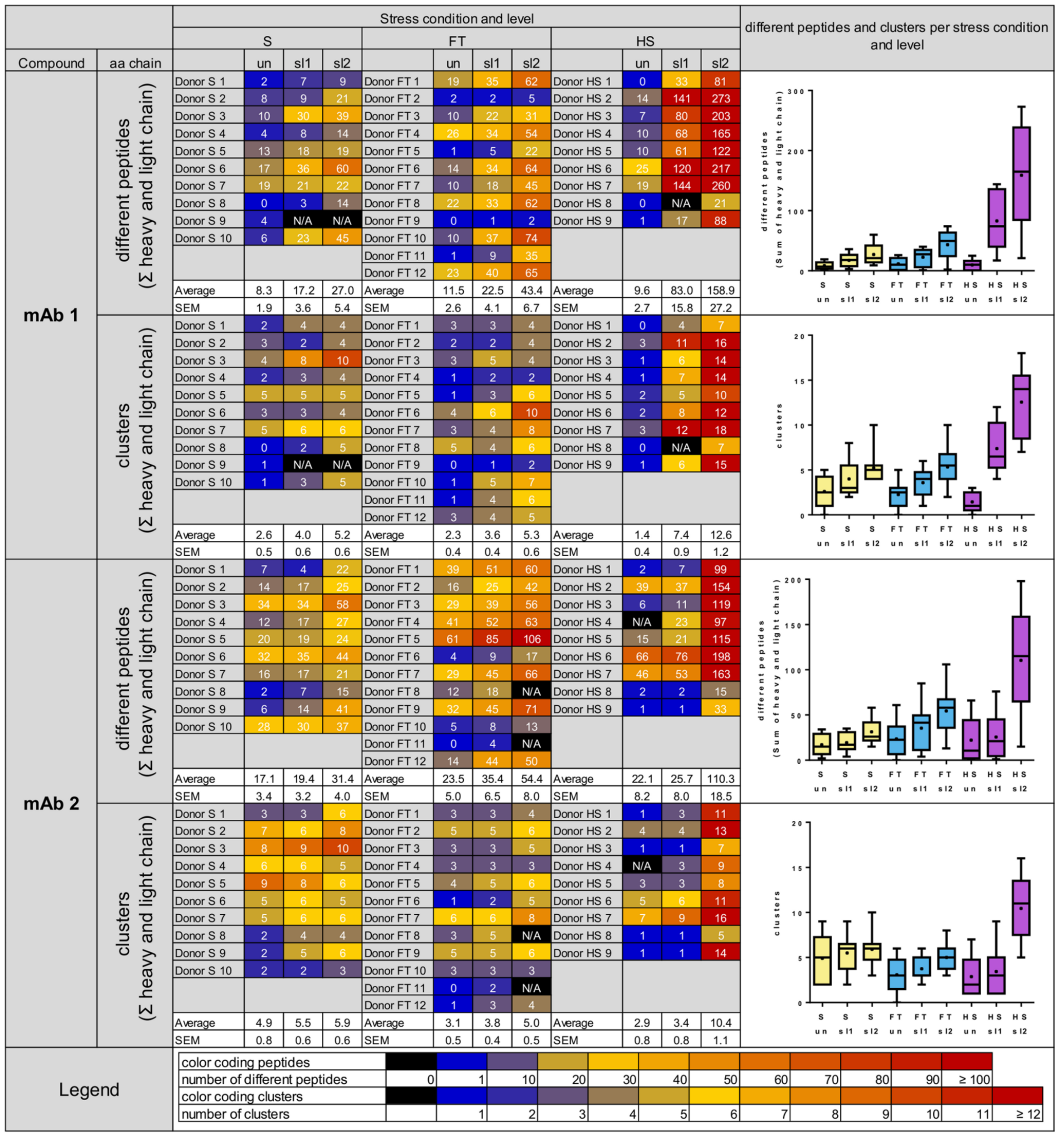
MAPPs results summary. Left: Heat map visualization of the number of different peptides and clusters (as sum of heavy and light chain) for both model antibodies mAb1 and mAb2 for all stress conditions S, FT and HS as well as stress levels un, sl1and sl2. Cell colors are reflecting the number of different peptides and clusters. Right: Box and whisker plot of the number of different peptides and clusters for the different mAbs, stress conditions and stress levels. The boxes extend from the 25th to 75th percentiles and the whiskers are plotted from the min to the max value. The median is indicated as a line and the mean as a dot within a box. HS: aggregates generated by heat and shake stress, FT: aggregates generated by freeze and thaw stress, S: aggregates generated by shear stress, mAb1: monoclonal antibody 1, mAb2: monoclonal antibody 2, un: unstressed, sl1: stress level 1, sl2: stress level 2.

**Figure 5:**
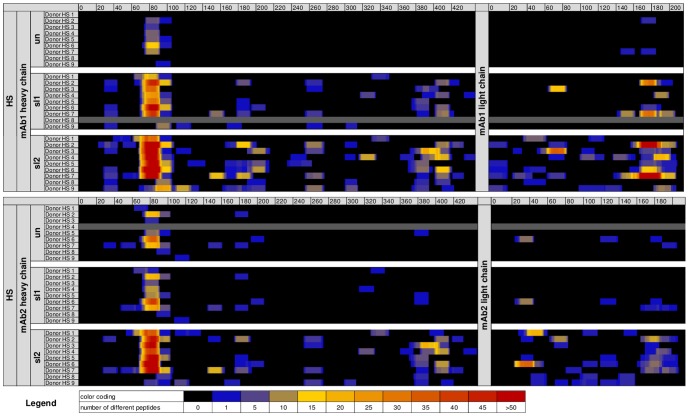
MAPPs heat map of identified HLA-DR associated peptides in the HS study. Heat map visualization of mAb-derived HLA-DR associated peptides for both model antibodies in the HS study. Each sequence position is colored according to the presence and number of different mAb-derived peptides identified. In black colored sequence regions, no peptides were identified, in colored regions, peptides were identified, with the color coding for the number of different peptides identified per position. HS: aggregates generated by heat and shake stress; mAb1: monoclonal antibody 1, mAb2: monoclonal antibody 2, un: unstressed, sl1: stress level 1, sl2: stress level 2.

In all samples derived from cells treated with model mAbs, peptides derived of the respective mAb could be identified (Figure 4). MAb-derived peptides clustered in several regions along the sequences of the antibody heavy and light chains (Figure 5 and Figure S6). For each stress condition, the average number of identified different peptides and clusters increased in samples incubated with stressed protein as compared to unstressed protein. Consistently for HS, FT and S stress, DCs incubated with sl2 material showed the highest number of different mAb-derived peptides and clusters. Upon stress, the mean increase of mAb-derived different peptides per donor ranged from 1.1 to 16.6-fold and the mean increase of mAb-derived clusters per donor from 1.1 to 8.7-fold over all stress conditions and levels (Table 1). The highest increase in the average number of different peptides and clusters per donor was detected for HS sl2, with a mean increase of different peptides of 16.6-fold and 5-fold for mAb1 and mAb2, respectively. The mean increase of clusters was 8.7-fold and 3.6-fold, for mAb1 and mAb2, respectively. A total of 20 clusters were identified for HS sl2 of mAb1 and 18 clusters for HS sl2 of mAb2 along the antibody heavy chains. In the antibody light chains, 9 clusters were identified for HS sl2 of mAb1 and mAb2 each. In summary, aggregation did not only change the frequency of presented peptides, but more importantly induced the presentation of new clusters in sequence regions not exposed to other immune cells under normal conditions.

Statistical analysis revealed that for mAb1 for all three stress conditions and all stress levels, the numbers of different peptides as well as clusters were significantly higher, as compared to DCs treated with unstressed material. For mAb2, the number of different peptides was significantly higher for FT and HS material at all stress levels as well as for S sl2 material, as compared to unstressed material. In addition, the number of clusters was significantly increased for FT sl2 and HS sl2 material when compared to unstressed material (Table 1).

#### Linear regression of the increase in number of different HLA-DR associated peptides and clusters as a function of the amount of protein contained in subvisible particles

**Figure 6:**
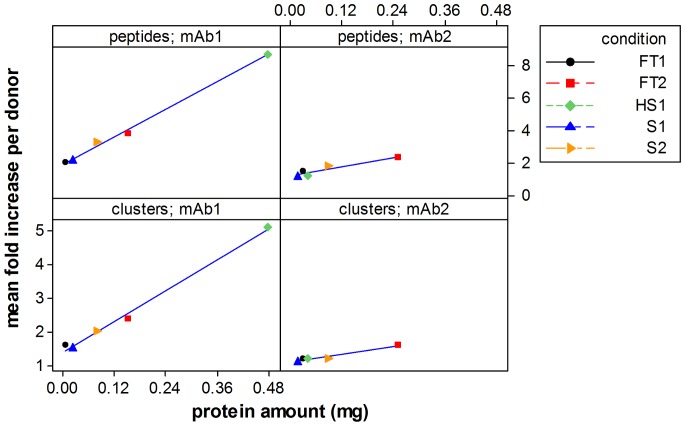
Correlation of HLA-DR associated peptides and peptide clusters measured by MAPPs to the amount of protein present in subvisible particles. Linear regression analyses of the increase of the HLA-DR associated peptides and clusters as functions of the calculated amount of protein present in the subvisible particles. Left up: HLA-DR associated peptides of mAb1 vs protein amount in subvisible particles (r^2^ = 0.994), left down: HLA-DR associated peptide clusters of mAb1 vs protein amount in subvisible particles (r^2^ = 0.993), right up: HLA-DR associated peptides of mAb2 vs protein amount in subvisible particles (r^2^ = 0.86), right down: HLA-DR associated peptide clusters of mAb2 vs protein amount in subvisible particles (r^2^ = 0.943). HS: aggregates generated by heat and shake stress; FT: aggregates generated by freeze and thaw stress, S: aggregates generated by shear stress mAb1: monoclonal antibody 1, mAb2: monoclonal antibody 2, 1: stress level 1, 2: stress level 2. For further details, please, see Materials and Methods.

In order to explore the possible causation of the changes in the HLA-DR associated peptides and clusters, their dependence on the amount of protein present in the subvisible particles in each condition was examined using linear regression analyses. The regression fits resulting from these analyses and the corresponding regression coefficients indicate that the increase of the amount of protein present in the subvisible particles can explain very well the increase of the number of peptides and clusters measured in the MAPPs assay (see Figure 6). Interestingly, the slopes of the regression lines obtained with this analysis differ substantially for mAb1 to mAb2 with mAb2 responses being generally lower.

### Activation of T-cells

**Figure 7:**
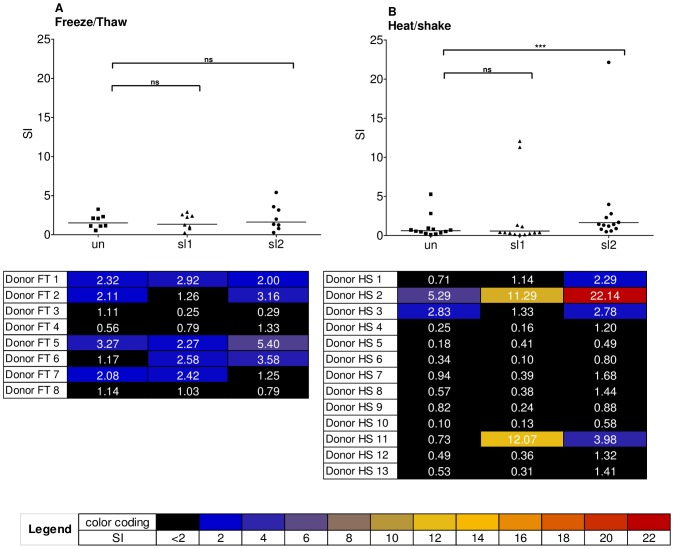
Results from T-cell activation assay. T-cell responses determined by IFN-γ ELISpot from donors treated with mAb1, un, sl1 and sl2 material from (A) FT condition (8 donors) and from (B) HS condition (13 donors). The respective heat maps specify to which extent each single donor responded to each treatment. The horizontal line shows the geometric mean of the populations. ***: statistically significant (p<0.001); ns: statistically not significant. HS: aggregates generated by heat and shake stress, FT: aggregates generated by freeze and thaw stress, mAb1: monoclonal antibody 1, SI: Stimulation index, un: unstressed, sl1: stress level 1, sl2: stress level 2.

In order to evaluate whether there is an increased activation of T-cells as a risk factor for immunogenicity in response to the aggregated antibodies, the unstressed and stressed mAb preparations were also analyzed in a T-cell study with a limited number of donors. T-cell activation was assessed by measuring IFN-γ release in an ELISpot assay in response to the same stressed material which was also used for the DC maturation and MAPPs assays using a 2-fold increase in the stimulation index (SI) as threshold for a positive response. Since mAb2 interfered with the T-cell activation assay due to its target-related mode of action, only mAb1 was used for this investigation. The tests included HS and FT stressed material at sl1 and sl2, and the unstressed mAb1. T-cell responses were analyzed in 8 healthy blood donors for FT conditions and in 13 different donors for HS conditions. In comparison to the donor set tested with the HS material, a higher proportion of the 8 donors tested with FT condition showed responses to unstressed mAb1 (Figure 7), demonstrating a higher responsiveness of this donor set as compared to the donor set used for HS material. However, the FT donor set showed only non-significant differences in the T-cell activation induced by unstressed *versus* stressed material for both sl1 and sl2 material.

Upon treatment with HS aggregated mAb1, T-cell activation was more prominent. While most of the donors did not respond at all to sl1, two donors, HS 2 and HS 11, showed very strong responses (SI = 11.29 and SI = 12.07, respectively), whereby donor HS 2 also showed a considerable response to the unstressed mAb1 (SI = 5.29). The response observed for donor HS 2 increased even further with sl2 material (SI = 22.14). In contrast, the response observed for donor HS 11 was less pronounced with sl2 material (SI = 3.98). Overall, only 2 donors responded to sl1, whereas 4 donors responded to sl2 material with SIs>2.

While the increase in the mean SI was statistically not significant for sl1 material, the signals of the whole donor set shifted in response to sl2 material, resulting in a highly significant increase of the mean SI (p = 0.0005), even when donor HS 2 was excluded from the statistical evaluation (p = 0.001).

## Discussion

The results from the *in vitro* studies presented here, measuring DC activation, T-cell activation and antigen presentation patterns in response to incubation with artificially stressed solutions of two model mAbs demonstrate that strongly aggregated mAbs are able to induce maturation of human monocyte-derived DCs. From all mAb solutions subjected to different types and levels of physical stress that were utilized in these studies, the material that induced strongest responses was the one that had undergone the highest levels of stress and nearly-completely aggregated (sl2). Intriguingly, the increase in the presentation of model mAb-derived HLA-DR associated peptides by DCs strongly correlated with the amount of protein contained within the subvisible particles. The effects of proteinaceous particles on DC activation and antigen presentation (MAPPs) were consistent with the capability of the aggregated material to induce IFN-γ secretion by T-cells. However, the effects on DCs observed in our *in vitro* experiments differ between the two model proteins which were used. This observation is consistent with previous studies employing orthogonal biological assays studying innate immune effects of aggregates which revealed differences in cytokine release observed for various mAbs analyzed under unstressed and highly stressed conditions in PBMC cultures [10].

The size distributions of the particles in all samples were similar, presenting a typical near-exponential decay, although the total particle counts naturally varied with the type and magnitude of each stress condition as well as the protein-intrinsic characteristics of each model mAb. Depending on the type of stress, the morphology of the induced aggregates differed considerably, with particles generated by freeze-thaw stress or shear stress showing dense spherical shapes in contrast to the filamentous particles induced by heat-shake stress. It is conceivable that such particle characteristics may influence the capacity to activate DCs and impact the processing of proteins by modulating the binding, uptake, enzymatic accessibility and peptide trimming [18], thereby contributing to different immunological responses *in vitro* and *in vivo*. Interestingly, a very strong correlation of antigen processing and presentation to the amount of protein that is contained within the subvisible particles was found.

Although the variability between response indices of individual donors in the DC assay was high for all tested stress conditions and levels, the differences in total particle counts in the aggregate preparations are reflected in their capacities to induce maturation of DCs *in vitro* i.e. more particles induce stronger DC maturation. Rather small average response indices for the different stress conditions and levels could be observed for mAb1. However, strong responses were found for HS sl2 material of mAb2. These findings indicate that intrinsic differences of mAbs may modulate the capacity to stimulate DC maturation. This is also illustrated by the fact that in this study unstressed, filtered material of mAb1 already showed a concentration-dependent level of DC maturation, while this effect was nearly absent for unstressed, filtered mAb2. The differences in DC maturation capacity and antigen presentation between mAb1 and mAb2 of both unstressed and stressed material underpin the importance of the primary and higher order structure of biopharmaceuticals with regard to responses of immune cells.

The mechanisms that could be involved in DC maturation and uptake of multimeric mAb complexes *in vivo* are multifaceted. It is well-described that monocyte-derived DCs express the Fcγ-receptors CD32a, CD16, CD32b – the first two being activating receptors, the latter one an inhibitory receptor, as well as complement receptors for C1q and iC3b [19]. One possibility is that aggregates of mAbs could increase the overall binding avidity of CD16 and CD32, which are rather ineffective in binding of monomeric mAbs due to their low affinity. *In vivo*, aggregates could also interact with C1q that restrictively binds to multimeric complexes and allows for activation of the complement cascade producing additional opsonizing components. It has been demonstrated that the activating Fcγ-receptors [20], [21], C1q [22] and other complement factors as well as downstream products of the complement cascade [23], [24] are effective mediators of DC maturation. An additional possibility is that misfolded proteins with exposed hydrophobic regions may be recognized by DAMP receptors and/or pattern recognition receptors (PPRs) including receptors of the TLR family that could trigger maturation of DCs [25]. Indeed, a contribution of TLR-2 and TLR-4 in aggregate-induced cytokine release from PBMCs has recently been demonstrated [11]. The *in vitro* system used here to analyze DC maturation by mAb aggregates did not take the contribution of complement-related mechanisms into account, since heat-inactivated fetal bovine serum (FBS) was used as supplement in the cultivation medium. Therefore, direct mAb-induced maturation capacity might be higher under comparable *in vivo* conditions. Also indirect mechanisms of DAMP production and liberation by tissue stress and damage *in vivo* [25], e.g. during local drug application, could not be covered in the assay, so that the presence of new or quantitatively increased T-cell epitopes might be sufficient to induce T-cell responses *in vivo* under certain conditions.

Besides activation of DCs by the potential mechanisms described above, the presence of strongly aggregated proteins could theoretically impact the normal response of the immune system by altering the presentation of these proteins by APCs. Using a unique and sensitive assay that allows for identifying HLA-DR associated peptides (MAPPs assay; [17]) directly from human DCs, this study has demonstrated for the first time that aggregation of model mAbs can have a strong influence on their presentation by DCs. It was established, that the number of different peptides and clusters presented were higher in samples treated with stressed mAb material as compared to the unstressed controls. Interestingly, material which was stressed more extensively generally resulted in stronger presentation of model mAb-derived peptides and clusters. Furthermore, an excellent linear relationship was observed between the amount of protein contained in the subvisible particles and the extent of presentation measured by the number of presented peptides or clusters (see Figure 4). The observed increase in the number of different peptides and clusters could be explained by different mechanisms: According to the “bind first – trim later” model [18] large fragments of the protein make contact with HLA-DR molecules and protruding parts are later trimmed by enzymes. By aggregating the protein, the change of the protein conformation could impact the accessibility of the aggregated protein to enzymes in the endolysosomal compartment, partially protecting the molecule from enzymatic degradation and preserving it for binding to HLA-DR molecules. Ultimately, this could result in an increase in the quantity of presented peptides as well as the emergence of neoepitopes. It has been shown that in DCs antigen uptake is limited to the first hours after maturation induction [26]. Thus, alternatively or in addition to a changed accessibility to enzymes, phagocytosis of large, micrometer-sized and densely compacted proteinaceous particles could lead to a rapid increase of the effective concentrations of that protein in the endolysosomal compartments as compared to taking up soluble material with far lower effective concentrations. Thereby, increased uptake of a protein could increase the quantity of the presented peptides derived from this protein by outcompeting self-peptides for binding to the limited number of HLA-DR molecules in the endolysosomal compartment. This could also be an explanation for the highest number of peptides and peptide clusters in samples treated with HS stressed mAb containing the highest amount of protein. It is also conceivable that neoepitopes could emerge by outcompeting self-peptides, thereby reaching the peptide density required for the activation of a T-cell. CD4 T-cell neoepitopes have a central role in the induction of adaptive immune responses, since the mature naïve T-cell repertoire may not have been confronted with these epitopes and, hence, induction of T-cell tolerance or depletion of reactive T-cells had not occurred *in vivo*. In addition, the density of a peptide on DCs is of high importance since a threshold density of a peptide is required to ensure the establishment of an effective immune synapse [27]. It has been shown that the density of a peptide determines the type of T-cell response induced [28] and it is conceivable that a strongly increased presentation of a peptide can switch the balance from a tolerogenic state towards an effector T-cell response.

An important detail which stands out and that could serve to interpret our findings in the context of the scenarios listed above is that the extent of the presentation correlates exceptionally well with the calculated amount of protein that is contained within the subvisible particles (particles larger than 2 µm). The straightforward linear relationship between amount of aggregated protein and antigen presentation observed in this study implies a major involvement of the mass-action mechanism, although an involvement of a changed enzymatic accessibility is still conceivable and could play a bigger role under different conditions and with different molecules. Attention needs to be called to the fact that in the results presented here, one of the factors of great importance determining the response was the primary structure of the protein, since in mAb1 experiments the increase of peptides and clusters was approximately 3-fold higher than in mAb2 experiments. Indeed, it is perfectly rational that the processes involved in antigen presentation by APCs (e.g. enzymatic cleavages and binding to the Ag-binding cleft of the HLA molecule) are sensitive to the individual protein (primary and higher order structure). The latter is evident by the different slopes of the relationship between MAPPs response (measured by either increase in peptides or clusters) and the protein amount that were obtained using mAb1 and mAb2. Coincidentally, an independent study was conducted by the company Antitope (Cambridge, UK) on characterized aggregates of monoclonal antibodies generated under various stress conditions that were evaluated on their potential to stimulate DC responses and to induce CD4 T-cell proliferation (personal communication M.P. Baker, Antitope). In alignment with our findings, these results indicate an influence of aggregates on DC activation and early CD4 T-cell priming.

Here we analyzed T-cell responses only for a small number of human donors without the intention to define a percentage of responders for each condition which would require a much larger set of donors as already published by others [10]. Responses were expected to only occur in a subset of the tested individuals, since these responses are dependent on the presence and reactivity of epitope-specific T-cells as well as on the two signals provided by the antigen presenting cells: (1) co-stimulatory molecules, which are only present on activated antigen presenting cells and (2) the peptides presented in the context of HLA class II molecules which are again dependent on the haplotype of each individual donor. Therefore, it is not surprising that a very strong increase in response to aggregates was only observed for a single donor (HS 2). It should also be noted that crude PBMCs were used for this assay and thus other cells than CD4 T cells may have contributed to the assay response. However, the strong increase in potential T helper cell epitopes as observed in the MAPPs assay indicates a major involvement of CD4 T cells. Marked increases in the peptide presentation were found in the MAPPs assay for all types of aggregates (S, FT and HS) and for both stress levels for mAb1, while significant T-cell responses were only observed for HS sl2 material. However, the MAPPs assay is independent of the induction of DC maturation by aggregates since maturation is artificially induced with LPS in order to maximize the peptide processing and presentation. Since, no artificial maturation stimulus was provided in the T-cell assay applied here, an increased activation of T-cells may only be expected for conditions leading to significant activation of antigen presenting cells, i.e. maturation of DCs. The observation that the mean SI significantly increased only in response to HS sl2 material is in line with results of the DC maturation assay, which showed strongest response indices and highest percentage of responding donors for HS-aggregated mAb1 at sl2 when compared to other stress conditions and levels for this model mAb. Moreover, also the alteration of peptide presentation profile was most pronounced for HS sl2, so that for all tested mAb1 conditions the two signals (co-stimulation and presence of a T-cell epitope) were highest in accordance with most effective CD4 T-cell activation.

Overall, these findings are in line with a recently published study in which increased adaptive CD4 T-cell responses to highly aggregated material were detected [10] and underpin the possibility that aggregates can provide both signals required for the induction of a T-cell response.

The phenomenon of protein aggregation is very complex with a number of parameters contributing to the aggregation propensity of a given protein. The two model molecules used in this study were selected on the basis of having very different biophysical properties from one another (isoelectric point (pI), melting temperature (T_m_) measured by DCS, surface hydrophobicity measured by hydrophobic exchange chromatography, colloidal stability - B_22_ measured by static light scattering), based on our extensive experience from the internal protein product pipeline. The results from our studies demonstrate that despite the differences in the intensity of responses in the different assays, the same general phenomena and trends are observed using both model proteins. This fact strongly underscores the general validity of our findings and in the same time highlights the importance of the primary structure and the intrinsic biophysical properties of proteins.

For the assessment of the effects of aggregates on DCs, the finite half-life and clearance of IgG in and from serum are an important aspect. The stability of subvisible particles in serum [29] and aggregate distribution *in vivo* [29] have been studied, suggesting that the aggregation profile and biodistribution may change upon administration. It has been found that particles formed by different stress methods can vary in their stability in serum ranging from 24 hours to several weeks, which underlines the complex and unpredictable behavior of protein aggregates in general [29]. However, antigen uptake by dendritic cells has been shown to be limited to the first hours after maturation induction [26], which indicates that uptake of aggregated material is likely to be faster than a potential change of the biodistribution and aggregation profile in serum. This observation is supported by the fact that the calculated amount of subvisible particles correlates very well with the presented peptides and clusters (Figure 6) independent of the type of stress which was applied.

A large amount of additional experimental work that involves a number of different proteins and stress conditions is needed in order to conclusively establish if the described mechanism is generic in the processing and presentation of aggregated proteins. However, the data presented here, convincingly demonstrate for the first time a fundamental difference in the presentation of aggregated as opposed to soluble proteins.

In summary, the *in vitro* data presented here suggest that in contrast to soluble mAbs, their aggregated suspensions can influence DC maturation, antigen presentation and activation of CD4 T-cells. Whereas T-cell activation by highly aggregated proteins has been demonstrated previously, the current report provides important additional evidence regarding the possibility for aggregates to activate monocyte-derived DCs, measured by the maturation marker CD83 and the T-cell co-stimulatory molecules CD80 and CD86. Most importantly, this study is unique in offering new details regarding the differences between the presentation of aggregated and soluble proteins. A unique assay (MAPPs) was used in order to interrogate the process of antigen presentation in DCs and the influence of aggregates on this process. Intriguingly, it was established that an apparent linear relationship exists between the amount of protein contained within the subvisible particles supplied to the simultaneously activated DCs and the increase in presentation of peptides and peptide clusters. Furthermore, this relationship was not majorly impacted by the type of physical stress exerted and the particle morphology. Although these findings were consistent using two different model monoclonal antibodies, the slopes of presentation increasing with the amount of aggregates were considerably different, revealing a major impact of the primary protein structure.

Aggregates and subvisible particles are present to a limited extent in every biopharmaceutical product sold on the market today. However, it is important to note, that the subvisible particles used in this mechanistic study were generated under exaggerated stress conditions and present in far greater concentrations than the ones typical for the currently marketed biotherapeutical antibodies. The translatability of these findings to the *in vivo* situation and their relevance to a broader range of diverse therapeutic proteins will require significant investments into investigative work in the future. Whether or not aggregate-provoked immune responses to therapeutic proteins occur also *in vivo*, still remains to be seen, however, the approaches presented here provide a valuable toolbox to further understand the mechanistic details of how the immune system manages aggregated proteins.

## Materials and Methods

The model antibodies (mAb1: 100 mg/ml and mAb2: 80.6 mg/ml) were diluted in DPBS (10× Dulbecco's, pH 6.5, Life Technologies Ltd, Paisley, UK) to 2 mg/ml. The protein solutions were sterile filtered (0.22 µm disposable, sterile MILLEX®GP filter units from Merck Millipore, Billerica, MA, USA) and aggregated by applying repetitive Freeze/Thaw cycles, shear stress, or heat/shaking stress. All working steps were performed in a sterile, particle free environment. Until final analysis all stressed samples were kept at −80°C.

### Generation of proteinaceous aggregates

#### Repetitive Freeze/Thaw cycles

4.5 ml of unstressed, diluted (2 mg/ml) and filtered protein solution were transferred into a 5 ml tube (Nalgene® Cryoware™, Nalge Nunc International Corporation, Rochester, NY, USA). Using a software program the samples were frozen and thawed automatically. Three (sl1) or ten (sl2) freeze/thaw cycles were performed. The samples were equilibrated to 4°C for 20 min before the first cycle. One cycle was defined as following: temperature decrease from 4°C to −40°C within 30 min, holding time at −40°C for 25 min, temperature increase from −40°C to 30°C within 35 min, followed by a holding time of 25 min at 30°C and finally, a temperature decrease from 30°C to 4°C within 5 min. For the very last cycle the temperature was hold at −40°C.

#### Shear Stress

1.5 ml aliquots were aggregated by drawing in and emptying each aliquot once (sl1) or four consecutive times (sl2) respectively using a disposable 5 ml silicone-free syringe (B. Braun Medical AG, Melsungen, Germany) with attached 19G×1½″ needle (BD). The aliquots were pooled in 250 ml Nalgene® bottles, mixed and split into 1 ml aliquots using 2 ml tubes (Nalgene® Cryoware™, Nalge Nunc International Corporation, Rochester, NY, USA).

#### Heat/shaking stress

1 ml aliquots (1.5 ml Eppendorf Biopur®, Eppendorf AG, Hamburg, Germany) were stressed for 10 min at 65°C and 1400 rpm (sl1) or 6 min at 80°C and 1400 rpm (sl 2) respectively. Before pooling and mixing all samples were cooled off to RT. Finally aliquots of 1.5 ml were prepared (2 ml tubes, Nalgene® Cryoware™, Nalge Nunc International Corporation, Rochester, NY, USA).

#### Thaw procedure

All stressed samples were thawed in a water bath at 38°C (4.5 ml: 30 min, 1 ml and 1.5 ml: 15 min) just before further analysis.

### Characterization methods

#### Micro Flow Imaging (MFI)

The MFI DPA4100 series A (Brightwell Technologies, Inc., Ontario, Canada) equipped with a 470 nm LED light source was used to detect and measure particles. For all pipetting steps filtered pipette tips from Mettler-Toledo International Inc. (Columbus, OH, USA) were used. The system was flushed with 15 ml HELLMANEX II (2%) solution (Hellma®, Hellma GmbH & Co. KG, Müllheim, Germany) and 15 ml water. For these flushing steps a 15 ml silanized glass syringe (Brightwell Technologies Inc., Ontario, Canada) was used. The flow rate was set to maximum speed. This flushing step was repeated after each different sample. 1 ml of unstressed sample was filtered (0.22 µm disposable, sterile MILLEX®GP filter units from Merck Millipore, Billerica, MA, USA) and was used to perform the “optimization of illumination” routine. Both, the unstressed, filtered protein and the stressed samples were gently mixed before 1 ml of each sample was measured. A maximum number of images were collected during each measurement.

#### Protein amount calculation

The equivalent circular diameter (diameter of a circle occupying the same pixel area as the actual pixel area) data reported from the Brightwell MFI software and an averaged protein density value of 1.43±0.03 mg/ml were used in order to calculate the estimated protein amount per size bin. Considering the water content using a correction factor of 0.75, integration of these masses, provides the total mass of the sample [30] water content was considered with a correction factor of 0.75.

#### Dynamic light scattering (DLS)

The Zetasizer nano ZS (Malvern Instruments GmbH, Herrenberg, Germany) equipped with a 633 nm He-Ne laser (4 mW) and a 633 nm Avalanche photodiode, positioned at 90° angle, for the detection of the scattered light was used to perform DLS. The instrument was switched on 30 min prior the analysis. 120 µl of each sample prepared as described above was measured in disposable UV-cuvettes micro (Plastibrand®, BRAND GmbH & Co. KG, Wertheim, Germany) at 25°C. Each measurement consisted of 20 runs. For the calculation of the Hydrodynamic radius 0.8872 mPa×s (η_water_, 25°C) was used. In the Zetasizer APS, the entire Gaussian distribution was used for the evaluation. The %PD value was ranked from 0% to 100%, describing the size distribution of the sample in the following way: PD%<25, predominantly monodisperse; PD% 25–40, monodisperse sample containing small amounts of polydisperse species and PD%>40, increasing polydispersity indicating presence of high MW species.

#### Size Exclusion Chomatography-Multi-Angle Laser Light Scattering (SEC-MALLS)

An Agilent 1100/1200 HPLC instrument (Agilent Technologies, Santa Clara, CA, USA) was connected to a laser light scattering detector (DAWN®TREOS® Wyatt Technology Corporation, Santa Barbara, CA, USA). Size exclusion chromatography was performed on a TSKgel G3000SW_XL_ analytical column (7.8×300 mm; TosohCorporation, Tokyo, Japan) at 30°C. The Mobile Phase was a 150 mM potassium phosphate buffer at pH 6.5. Sample vials were kept at 4°C. 150 µg of protein prepared as described above was injected and analyzed at a flow rate of 0.4 ml/min. The UV absorbance was measured at 280 nm.

#### Capillary Electrophoresis-Sodium Dodecyl Sulfate (CE-SDS)

CE-SDS was performed both under reducing, and non-reducing conditions.

Under reducing conditions, samples were denatured with SDS and β-mercaptoethanol for 10 min at 70°C, under non-reducing conditions, samples were denatured with SDS and alkylated with Iodoacetamide for 10 min at 70°C before injection in a Beckman Coulter Capillary Electrophoresis System ProteomeLab PA800 plus (Beckman Coulter Inc, Fullerton, CA, USA). The capillary was rinsed at 70 psi with 0.1 mM NaOH for 5 min and then with 0.1 mM HCl for 5 min and deionized water for 1.0 min. The SDSMW gel Buffer was loaded into the capillary at 70 psi for 10 min. Samples were injected electrokinetically at 5 kV for 30 s from the inlet for reducing and from the outlet for non-reducing conditions. The separation was performed at −15 kV (−500 V/cm) for reducing and 7.5 kV (250 V/cm) for non-reducing conditions, maintaining the capillary temperature at 25°C followed by UV detection at 214 nm.

#### Peptide mapping

Lys-C digestion: 200 µg of protein samples were denatured using 150 µl of denaturing solution (6 M guanidine hydrochloride, 50 mM Tris-HCl, 5 mM Na_2_EDTA, pH 8.0), and reduced by adding 1.5 µl of 1 M DTT. The reduction was performed for an hour at 37°C. The alkylation was performed by adding 3 µl of 1 M iodoacetamide followed by incubation at room temperature in the dark for an hour. The reaction was quenched with 1 µl of 1 M DTT. Following reduction/alkylation, 750 µl digestion buffer (50 mM Tris-HCl, pH 8) was added to the sample. The sample was further digested with two additions of 4 µl Endoproteinase Lys-C (1 mg/ml, Wako (Osaka, Japan)) at 37°C incubation for 1 hour and 3 hours respectively. 5 µl TFA was added to stop the digestion.

Instrumentation: Lys-C digested peptides were subjected to LC-MS/MS (liquid chromatography-mass spectrometry) using an Agilent 1200 series HPLC system equipped with a diode-array detector and coupled to a LTQ Velos ion trap mass spectrometer (Thermo Fisher Scientific, Hemel Hempstead, UK). Reversed-phase chromatography separation was carried out on a 2.1×150 mm Vydac C18 column packed with 5 µm particles, 300 Å pore size (Grace, Deerfield, IL, USA). The eluents were A: 0.1% TFA in water and B: 0.09% TFA in acetonitrile. The column was set at 40°C and the flow rate was 200 µL/min. Samples were eluted with 0% B (0.00–5 min); 0 to 2% B (5–10 min); 2–5% B (10–15 min); 5–20% B (15–60 min); 20–22% B (60–95 min); 22–33% B (95–130 min); 33–35% B (130–150 min); 35–70% B (150–175 min); 70–100% B (175–180 min).

For MS and MS/MS analysis, the ESI voltage was set at 4 kV in positive ion mode. The capillary temperature was 275°C. One full scan mass spectrum was followed by MS/MS scans of the three most intense ions. MS/MS data were scanned in enhanced mode and the isolation width was 3.0 Da, the collision energy was 35%, and the activation time was 10 milliseconds.

Evaluation: Peptides were identified manually by comparing the observed peptide mass with the theoretical peptide mass generated from the known amino acid sequence using the GPMAW software.

#### Cross-reactivity testing

The cross-reactivity profiles of all antibody preparations were determined using the protein microarray UNIchip® AV-VAR EP (Protagen AG, Dortmund, Germany). The chips contain 403 pre-defined extracellular and secretory proteins. The hybridization of the antibodies was done using a TECAN Hybridization Station HS 4800™ Pro (Tecan Group Ltd., Männedorf, Switzerland) and an incubation program developed by Protagen AG. Microarray image acquisition was performed employing a TECAN PowerScanner™ fluorescence microarray scanner. Image analysis was accomplished using the GenePix Pro v7.0 software. Protagen UNIchip® Data Analysis Tool v2.2.5 was used to analyze the data. To determine unspecific cross-reactivity derived from binding of the detection antibody to the printed proteins, the detection antibody was also incubated on the UNIchip® without the use of a primary antibody. An isotype control human IgG was used to normalize the level of cross-reactivity. Proteins with a relative signal equal or greater than 4% (cut-off corresponds to m+3σ measured on the background) were considered as positive hits, providing they were not found in the control chips incubated with only the detection antibody.

### In vitro DC Assay

#### Isolation of monocytes

Isolation of peripheral mononuclear blood cells (PBMCs). PBMCs were isolated from human buffy coats sampled from consent healthy donors (Blood Donation Center SRK beider Basel) according to local ethical practice. PBMC isolation was achieved by density gradient centrifugation using Leucosep tubes (Greiner Bio One, Frickenhausen, Germany) according to manufactures recommendations.

The isolated PBMCs were labeled with human anti-CD14 microbeads (Miltenyi Biotech, Bergisch Gladbach, Germany) according to the manufacturer's protocol. After filtration of labeled cells through pre-separation filters (Miltenyi Biotech, Bergisch Gladbach, Germany), cells were magnetically separated with LS MACS columns in a magnetic field. Purity of monocytes determined via flow cytometry after surface staining was consistently above 90% CD14+ cells.

#### Differentiation to immature dendritic cells

After viability confirmation with Trypan blue (Sigma-Aldrich, Buchs, Switzerland) purified monocytes were resuspended in warm medium (RPMI1640; 10% FCS; 1% L-Glutamine, 1% HEPES, 1% non-essential amino acids, 1% Penicillin/Streptomycin) containing 1000 U/ml granulocyte monocyte-colony stimulating factor (GM-CSF; Miltenyi Biotech, Bergisch Gladbach, Germany) and 1000 U/ml interleukin-4 (IL-4; Miltenyi Biotech, Bergisch Gladbach, Germany) to a final cell concentration of 2.5×10^6^ cells/ml and differentiated to immature DCs for 5 days at 37°C and 5% CO_2_.

#### Maturation of dendritic cells

After medium exchange immature DCs were seeded into 24 well cell culture plates (Nunc, Langenselbold, Germany) containing 1×10^6^ cells/ml/well. Before use in the assay, the aggregated materials and the unstressed mAbs were tested by Limulus amebocyte lysate test to exclude any lipopolysaccharide interference with the assay. The test revealed endotoxin levels below the detection level of the assay of 0.25 EU/mg protein. Any interference could therefore be excluded. Aggregated drug material (and corresponding controls in DPBS), i.e. unstressed filtered drug, were added at 200, 70 and 10 µg/ml not exceeding a final volume proportion of 10% in culture (100 µl/well). The formulation buffer DPBS was included as negative control (100 µl/well) in addition to a control in medium only. A cytokine cocktail containing 1000 U/ml GM-CSF, IL-4, IL-6 (Biolegend, Fell, Germany), IL-1β (Biolegend, Fell, Germany), tumor necrosis factor-alpha (TNF-α; Roche Applied Science, Rotkreuz, Switzerland) and 1 µg/ml prostagladine E2 (PGE2; Sigma-Aldrich, Buchs, Switzerland) served as a strong positive maturation control. Maturation was done over 68 h–72 h in an incubator at 37°C and 5% CO_2_. All conditions were tested in single wells as low inter-well variability was shown in preceding experiments.

#### Surface staining

In order to prevent unspecific binding of antibodies a 15 min blocking step with 100% FBS (heat inactivated, Gibco, Life Technologies, Zug, Switzerland) was performed before staining. Staining was done at optimally titrated concentrations with mouse anti-human antibodies specific for surface markers of interest - CD83-FITC, CD86-PE, CD80-PC7, CD14-ECD, CD11c-PC5 (all purchased from Beckman Coulter, Nyon, Switzerland) - and with corresponding isotype control antibodies for 20 min at 4°C in the dark. In order to remove unbound antibodies, cells were washed once with FACS buffer (PBS+1.0% FBS+0.05% sodium azide) before being finally re-suspended in FACS buffer containing fixative (Beckman Coulter, Nyon, Switzerland). Fixed cells were stored at 4°C in the dark until measurement.

#### Multicolor flow cytometry-based analysis

Measurement was conducted on the FC500MPL flow cytometer (Beckman Coulter, Nyon, Switzerland) using MXP acquisition software. The cut off for data acquisition was 10.000 events of gated DCs. For data evaluation Kaluza software (Version 1.1; Beckman Coulter, Nyon, Switzerland) was applied. Isotype controls were used to set the threshold for the specific staining. Mean fluorescence intensity (MFI) of maturation markers was assessed on CD11c+ cells.

### MHC associated Peptide Proteomics assay (MAPPs assay)

#### Generation of HLA-DR specific beads for immunoprecipitiation

Monoclonal antibodies specific for HLA-DR molecules were generated using the mouse hybridoma cell line L243 [29]. Protein A purified anti-HLA-DR antibody was immobilized on NHS-activated beads (GE Healthcare Bio-Sciences AB, Uppsala, Sweden) according to the manufacturer's protocol and stored containing 0.02% sodium azide. For confirmation of HLA-DR depletion efficiency of the L243-conjugated beads, cell lysates before and after immunoprecipitation with the beads were analyzed by Western Blotting using the HLA-DR-specific mAb 1B5 (Lifespan Biosciences, Seattle, WA, USA)

#### Isolation of monocytes

Monocytes were isolated as described in the DC assay method section.

#### Differentiation of monocytes to immature DCs

Monocytes were re-suspended in warm medium (RPMI 1640 w/o Glutamine; 10% FCS; 1% Glutamax; 1% non-essential amino acids, 1% Sodium-pyruvate, 1% kanamycine) containing 33 ng/ml granulocyte monocyte-colony stimulating factor (GM-CSF; Miltenyi Biotech, Bergisch Gladbach, Germany) and 3 ng/ml interleukin-4 (IL-4; Miltenyi Biotech, Bergisch Gladbach, Germany) to a final cell concentration of 0.3×10^5^ cells/ml and differentiated to immature DCs in cell culture dishes for 5 days at 37°C and 5% CO_2_.

#### Stimulation and loading of immature DCs

On day 5 of cell culture, aggregate preparations were thawed for 15 min (S) or 30 min (FT and HS) in a water bath at 38°C. Immature DCs were induced to maturation by adding LPS (1 mg/ml, Sigma, St. Louis, MO, USA) and simultaneously loaded with the different aggregate- preparations at a concentration of 70 µg/ml. Unloaded DCs served as negative control. After incubation for 24 hours at 37°C and 5% CO_2_, DCs were harvested and washed in PBS. After removal of liquid, the cell pellets were frozen at −70°C.

#### Isolation of HLA-DR associated peptides

For isolation of HLA-DR associated peptides, DC pellets obtained from 5×10^6^ cells were lysed in hypotonic buffer containing 1% Triton X-100 and protease inhibitor tablets (Roche Diagnostics GmbH, Mannheim, Germany) for 1 hour at 4°C on a horizontal shaker at 1100 rpm. After centrifugation, the lysate was incubated over night at 4°C with L243-conjugated beads for immunoprecipitation. After washing with wash buffer (PBS containing 0.5% Zwittergent) and several wash steps with distilled water, peptides were eluted from HLA-DR molecules by adding 0.1% trifluoracetic acid (Fluka, Buchs, Switzerland) at 37°C and lyophilized using an Eppendorf Concentrator 5301(Eppendorf AG, Hamburg, Germany).

#### Analysis of HLA-DR associated peptides

Lyophilized peptides were resuspended in hydrophilic buffer containing 5% acetonitrile, 0.012% heptafluorbutyric acid and 1.1% formic acid and injected onto a self-packed fused-silica C18 reversed phase nano-HPLC column. Multiple injections were not performed due to limited amounts of sample. Peptide identification was performed using liquid chromatography (nano capillary system, Dionex Corporation, Sunnyvale, CA, USA) on a reversed phase column connected to a mass spectrometer (LTQ Velos Orbitrap, Thermo, CA, USA) via electrospray ionization (LC-ESI-MS/MS). Peptides were identified using a database search approach using the SEQUEST algorithm. Peptides with a delta mass of <5 ppm to the expected mass, cross correlation values of XCorr >1.8 for singly charged ions, >2.3 for doubly charged ions and >2.8 for triply charged ions and a delta cross correlation (dCn)>0.1 were considered as true hits. In model mAb treated samples, mAb-derived peptides were detected multiple times and in several length variants clustering in sequence regions increasing the confidence in correctness of peptide identification. In addition donors sharing the same HLA-DR alleles showed common peptide clusters.

Lack of identification of model mAb-derived peptides in a negative control samples, ruled out false positive identification of model mAb-derived peptides in the respective samples derived from the same donor.

Peptides with differing amino acid composition exhibit different flight characteristics in electrostatic and electrodynamic fields within the mass spectrometer, thereby impacting the detection efficiency of different peptides and rendering quantification of peptides a challenge. However, in our study samples derived from the same donor showed variation in the numbers of different peptides below 12%. This implies comparable peptide distribution in the sample as well as comparable peptide loading efficiency and recovery, allowing for comparison of the number of model-mAb derived different peptides and clusters within donors.

T-cell assayPBMCs were isolated as described in the in-vitro DC assay section. After isolation, PBMCs were resuspended in Ex-Vivo medium (BE04-418Q, Lonza, Basel, Switzerland) at 4–6×10^6^ cells/ml and plated in 12 well plates with 1 ml/well on day 0. Aggregate preparations of mAb1 were added to the PBMCs at a final concentration of 200 µg/ml. On day 1 another 1 ml of medium was added to each well. Cultures were incubated at 37°C and 5% CO_2_. After 7 days, cells were harvested and the cell concentrations were readjusted to 4–6×10^6^ cells/ml. Then 200,000 cells were transferred to each well of a 96-well filter plate to determine the number of IFN-γ secreting cells by enzyme linked immuno spot assay (ELISpot, Human IFN-γ ELISpot kit, 3420-4APT-4, Mabtech AB, Sweden). Samples were measured in duplicates. An anti-CD3 antibody and PHA (2.5 µg/ml) were used as positive controls. The ELISpot plates were incubated for 16–18 hours in humidified incubator at 37°C and 5% CO_2_. The ELISpot plates were then developed according to the manufacturer's protocol. The spots were counted using an automated ELISpot reader system (Analyzer S5 Core Immunospot, Cellular Technology, C.T.L. Shaker Heights, OH, USA), using Immunospot analysis software. The SI was calculated by the ratio between the spot count of the treated cells and the spot count of the non-treated control.

### Statistical analyses

To evaluate the statistical significance of differences in peptide and cluster presentation in the MAPPs assay and in the expression levels of maturation markers in the DC assay obtained for unstressed versus stressed mAb materials, the different numbers of peptides and clusters in both heavy and light chain (MAPPs assay) and the mean fluorescence intensities for the different maturation markers (DC assay) were analysed by paired, two-sided Student's t-test by GraphPad Prism 5.03 Software for Windows (La Jolla, CA, USA).

Correlation analyses for the DC assay were performed between total particle counts and the average response indices of all three maturation markers (average x-fold upregulation of overall marker expression) In these correlations, the HS2 mAb1 and mAb2 results could not be considered, since the particle counts were above the MFI detection limit. The Spearman rank correlation coefficient for analysis of trend associations was calculated using OriginLab data analysis and graphing software (Northhampton, MA, USA).

The linear regression analyses for the increases in HLA-DR associated peptides and peptide clusters were performed using Minitab 15.0 statistical software (State College, PA, USA). The protein amounts contained in subvisible particles were calculated as described above.

The T-cell responses observed in the T-cell assay were not normally distributed. Therefore, to compare the mean stimulation indices observed in response to the different stressed mAb materials the Wilcoxon matched-pairs test (two-tailed) was applied using GraphPad Prism 6.02 Software for Windows (La Jolla, CA, USA).

## Supporting Information

Figure S1 **Non-reduced and reduced CE-SDS electropherograms of (A and C) mAb1 and (B and D) mAb2 of HS stressed material.** Color code: un (black), sl1 (red), sl2 (blue). HS: aggregates generated by heat and shake stress, mAb1: monoclonal antibody 1, mAb2: monoclonal antibody 2, un: unstressed, sl1: stress level 1, sl2: stress level 2.
(TIF)

Figure S2 **Non-reduced and reduced CE-SDS electropherograms of (A and C) mAb1 and (B and D) mAb2 of FT stressed material.** Color code: un (black), sl1 (red), sl2 (blue). FT: aggregates generated by freeze and thaw stress, mAb1: monoclonal antibody 1, mAb2: monoclonal antibody 2, un: unstressed, sl1: stress level 1, sl2: stress level 2.
(TIF)

Figure S3 **Non-reduced and reduced CE-SDS electropherograms of (A and C) mAb1 and (B and D) mAb2 of S stressed material.** Color code: un (black), sl1 (red), sl2 (blue). S: aggregates generated by shear stress, mAb1: monoclonal antibody 1, mAb2: monoclonal antibody 2, un: unstressed, sl1: stress level 1, sl2: stress level 2.
(TIF)

Figure S4 **LC/MS-peptide map chromatograms at 214 nm of mAb1-(A):FT, (B):S, (C):HS and mAb2-(D):FT, (E):S, (F):HS stressed samples.** The corresponding aggregation levels are displayed in blue (un), red (sl1) and green (sl2). HS: aggregates generated by heat and shake stress, FT: aggregates generated by freeze and thaw stress, S: aggregates generated by shear stress, mAb1: monoclonal antibody 1, mAb2: monoclonal antibody 2, un: unstressed, sl1: stress level 1, sl2: stress level 2.
(TIF)

Figure S5 **Overlay of SEC chromatograms of unstressed protein (black), stress level 1 (red) and stress level 2 (blue) material.** Chromatograms are shown for (A) mAb1 HS, (B) mAb1 FT, (C) mAb1 S, (D) mAb2 HS, (E) mAb2 FT and (F) mAb2 S. HS: aggregates generated by heat and shake stress, FT: aggregates generated by freeze and thaw stress, S: aggregates generated by shear stress, mAb1: monoclonal antibody 1, mAb2: monoclonal antibody 2.
(TIF)

Figure S6 **MAPPs heat map of identified HLA-DR associated peptides.** Heat map visualization of mAb-derived HLA-DR associated peptides for both model antibodies. Each sequence position is colored according to the presence and number of different mAb-derived peptides identified. HS: aggregates generated by heat and shake stress, FT: aggregates generated by freeze and thaw stress, S: aggregates generated by shear stress, mAb1: monoclonal antibody 1, mAb2: monoclonal antibody 2, un: unstressed, sl1: stress level 1, sl2: stress level 2.
(TIF)

Table S1 **HLA DRB1 haplotypes of donors tested in MAPPs assay.**
(DOCX)
