# Correction: Aggregation of Human Recombinant Monoclonal Antibodies Influences the Capacity of Dendritic Cells to Stimulate Adaptive T-Cell Responses *In Vitro*

**DOI:** 10.1371/journal.pone.0093339

**Published:** 2014-03-19

**Authors:** The PLOS ONE Staff

In the Results, the second sentence of the section entitled “Increase of model antibody-derived different peptides,” should list units of LPS as μg/ml. In the Materials and Methods, the second sentence of the section entitled “Stimulation and loading of immature DCs” should also list the units of LPS as μg/ml.
