# Supplementary figures and images for: Aggregation of Human Recombinant Monoclonal Antibodies Influences the Capacity of Dendritic Cells to Stimulate Adaptive T-Cell Responses In Vitro
Source: PLoS One. 2014 Jan 21;9(1):e86322. doi: 10.1371/journal.pone.0086322 (PMC3897673; doi:10.1371/journal.pone.0086322)

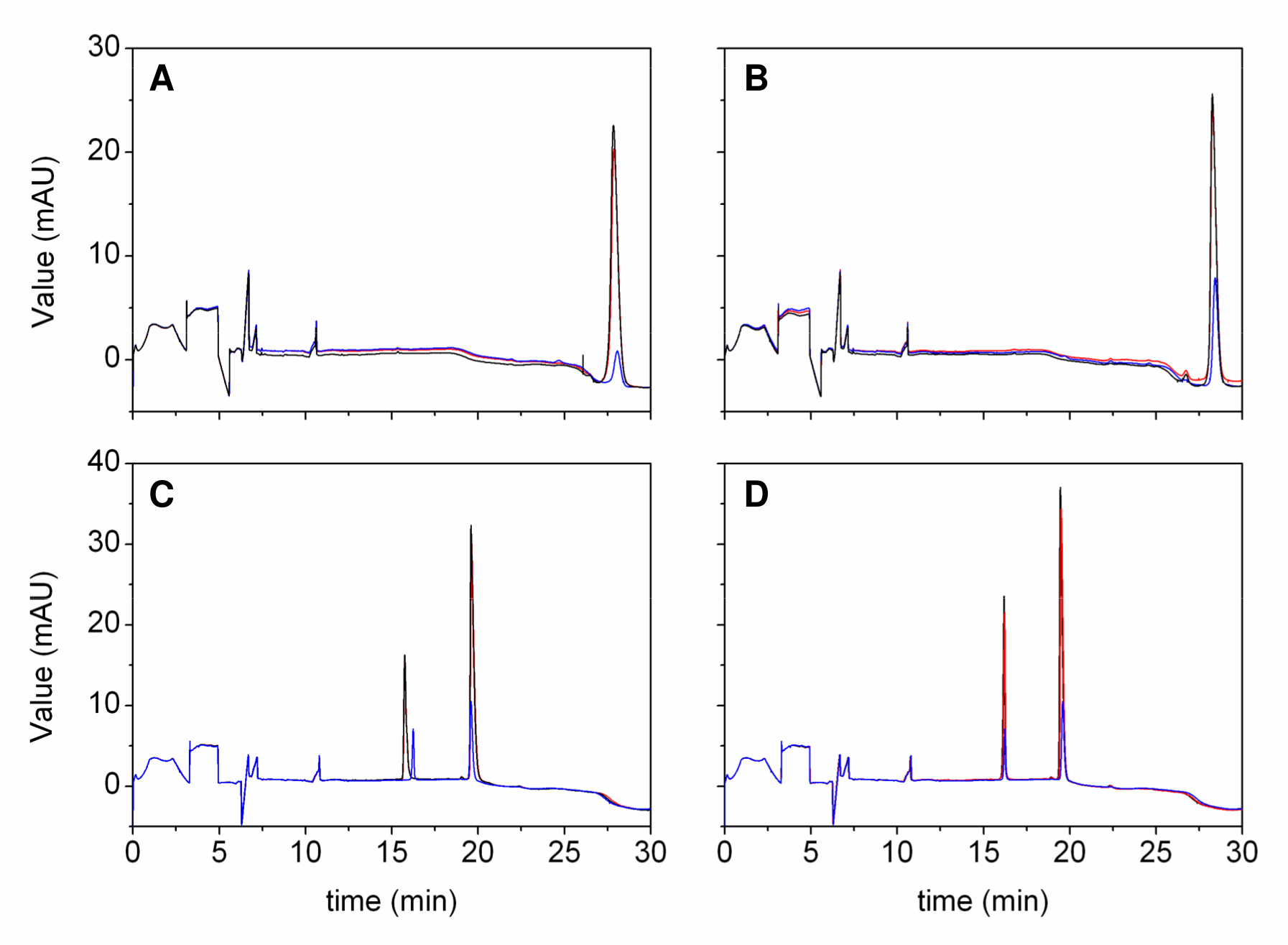

Supplement: Figure S1 — Non-reduced and reduced CE-SDS electropherograms of (A and C) mAb1 and (B and D) mAb2 of HS stressed material. Color code: un (black), sl1 (red), sl2 (blue). HS: aggregates generated by heat and shake stress, mAb1: monoclonal antibody 1, mAb2: monoclonal antibody 2, un: unstressed, sl1: stress level 1, sl2: stress level 2. (TIF) [file pone.0086322.s001.tif]

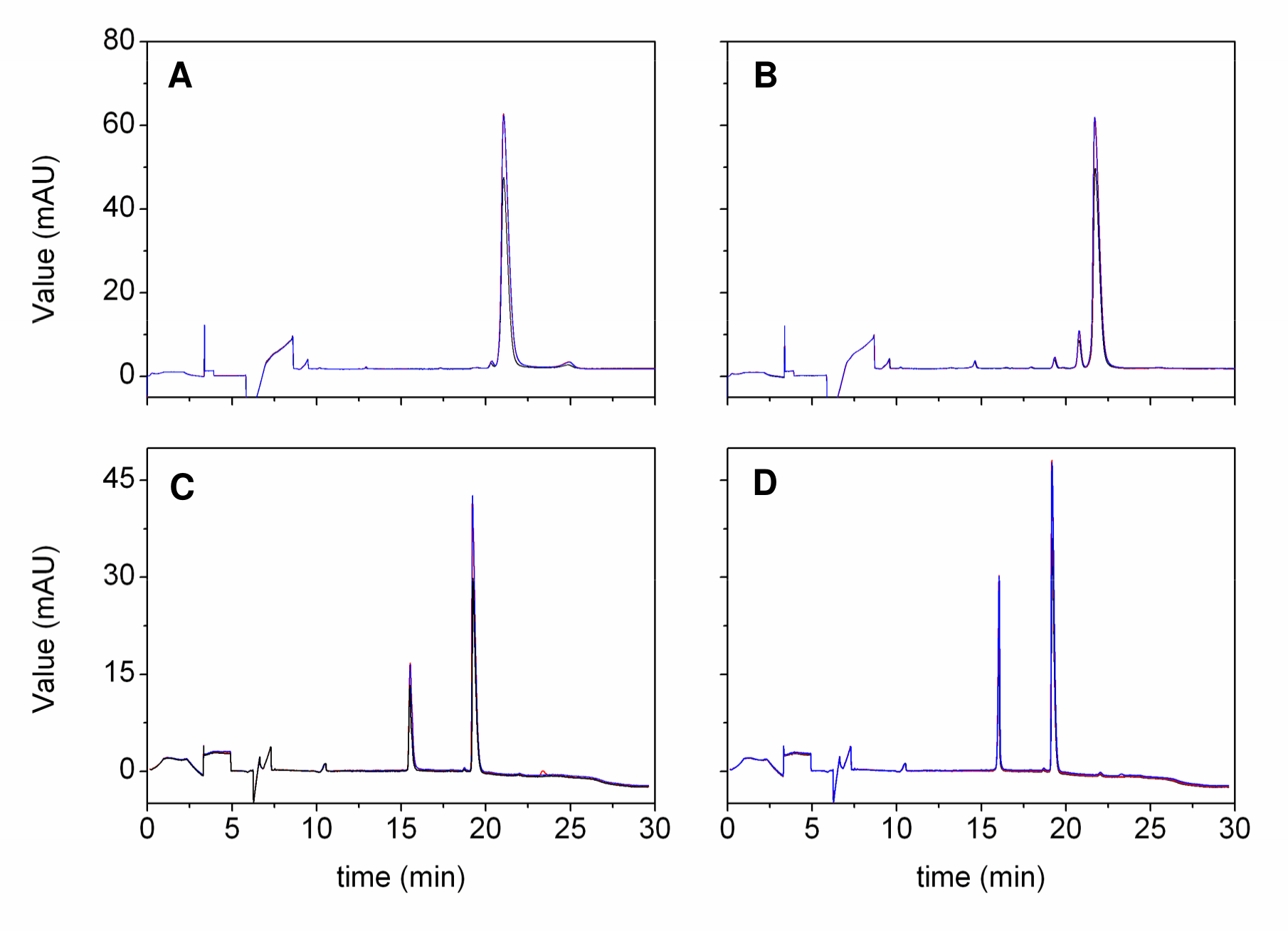

Supplement: Figure S2 — Non-reduced and reduced CE-SDS electropherograms of (A and C) mAb1 and (B and D) mAb2 of FT stressed material. Color code: un (black), sl1 (red), sl2 (blue). FT: aggregates generated by freeze and thaw stress, mAb1: monoclonal antibody 1, mAb2: monoclonal antibody 2, un: unstressed, sl1: stress level 1, sl2: stress level 2. (TIF) [file pone.0086322.s002.tif]

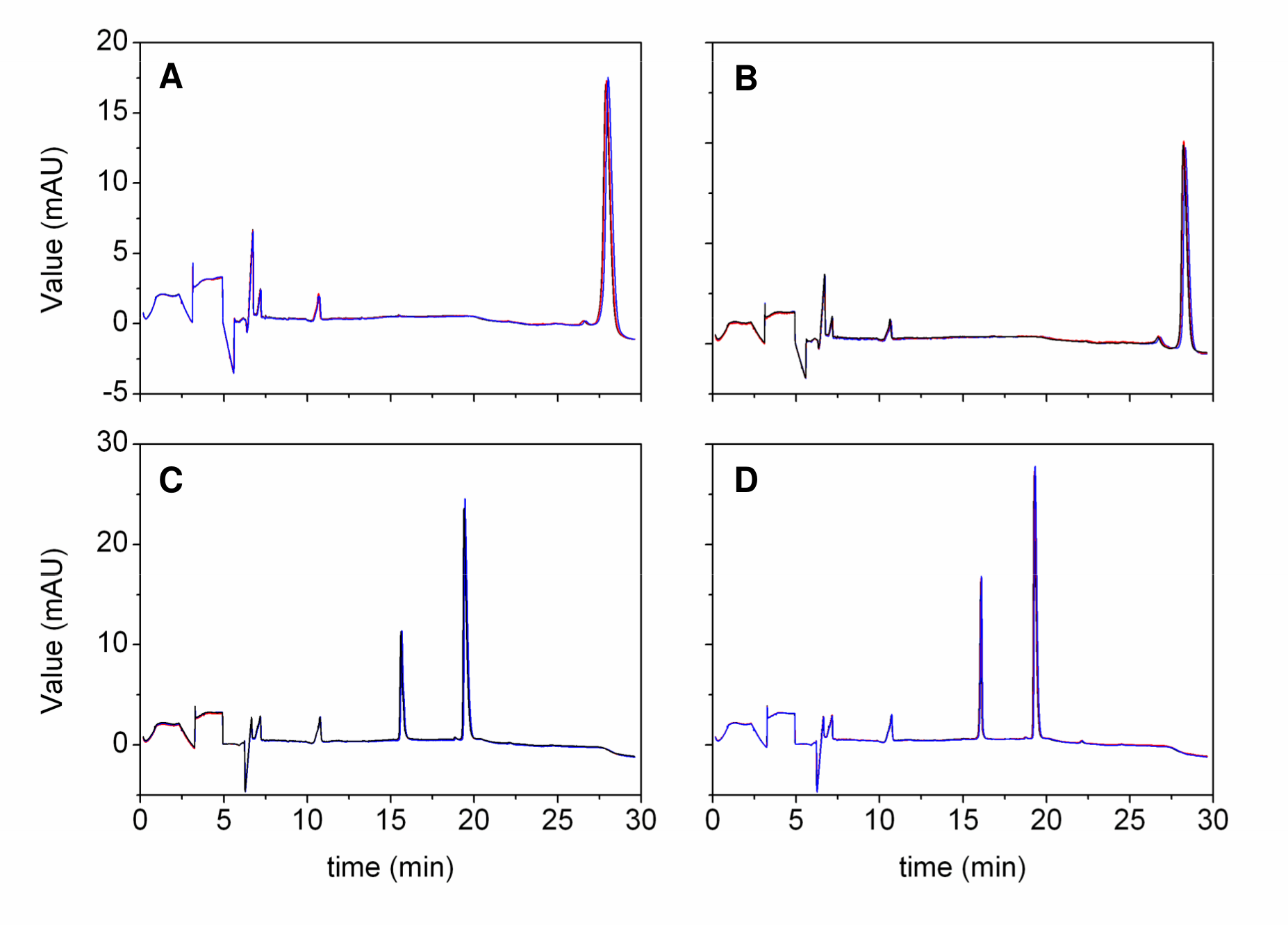

Supplement: Figure S3 — Non-reduced and reduced CE-SDS electropherograms of (A and C) mAb1 and (B and D) mAb2 of S stressed material. Color code: un (black), sl1 (red), sl2 (blue). S: aggregates generated by shear stress, mAb1: monoclonal antibody 1, mAb2: monoclonal antibody 2, un: unstressed, sl1: stress level 1, sl2: stress level 2. (TIF) [file pone.0086322.s003.tif]

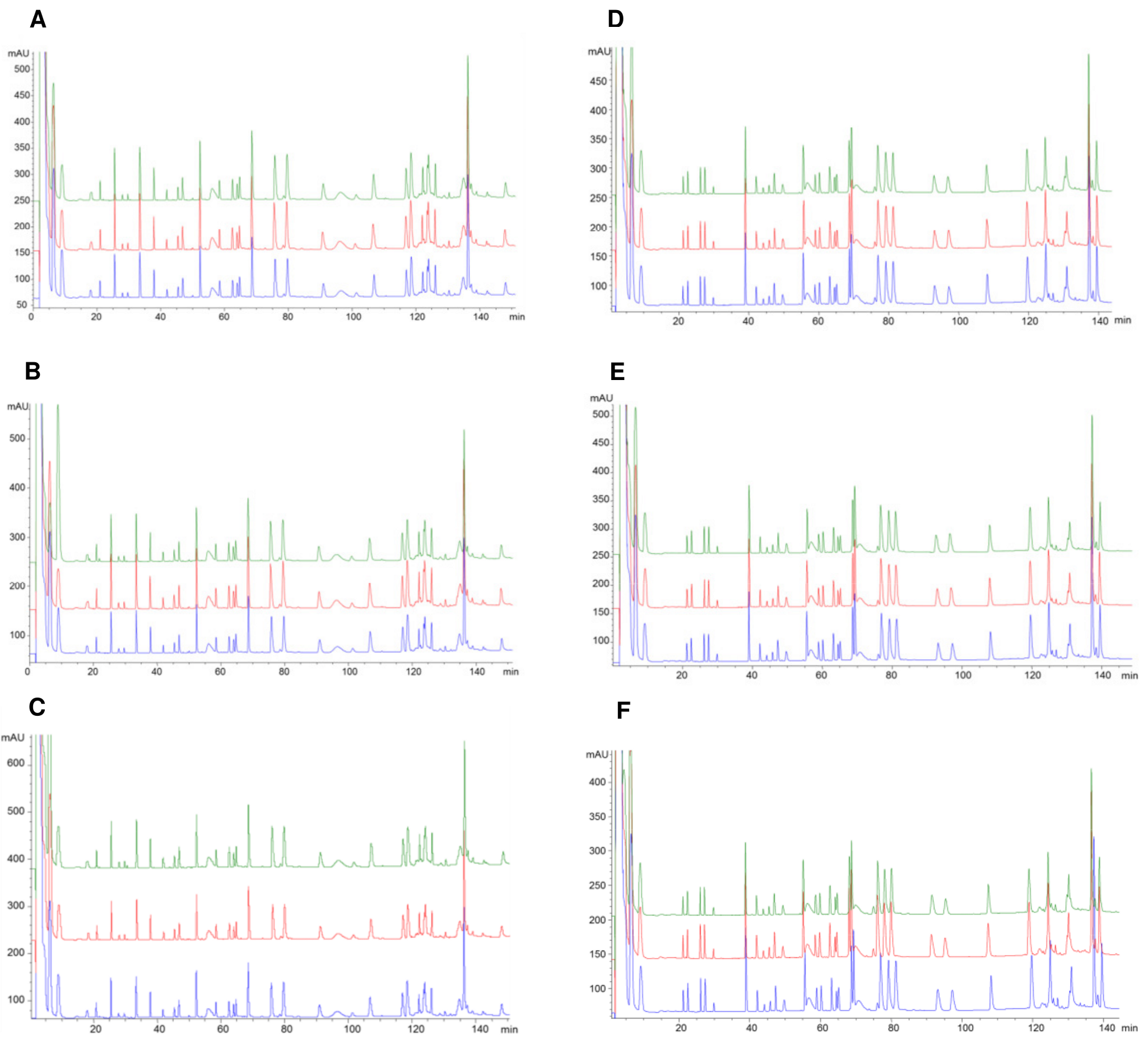

Supplement: Figure S4 — LC/MS-peptide map chromatograms at 214 nm of mAb1-(A):FT, (B):S, (C):HS and mAb2-(D):FT, (E):S, (F):HS stressed samples. The corresponding aggregation levels are displayed in blue (un), red (sl1) and green (sl2). HS: aggregates generated by heat and shake stress, FT: aggregates generated by freeze and thaw stress, S: aggregates generated by shear stress, mAb1: monoclonal antibody 1, mAb2: monoclonal antibody 2, un: unstressed, sl1: stress level 1, sl2: stress level 2. (TIF) [file pone.0086322.s004.tif]

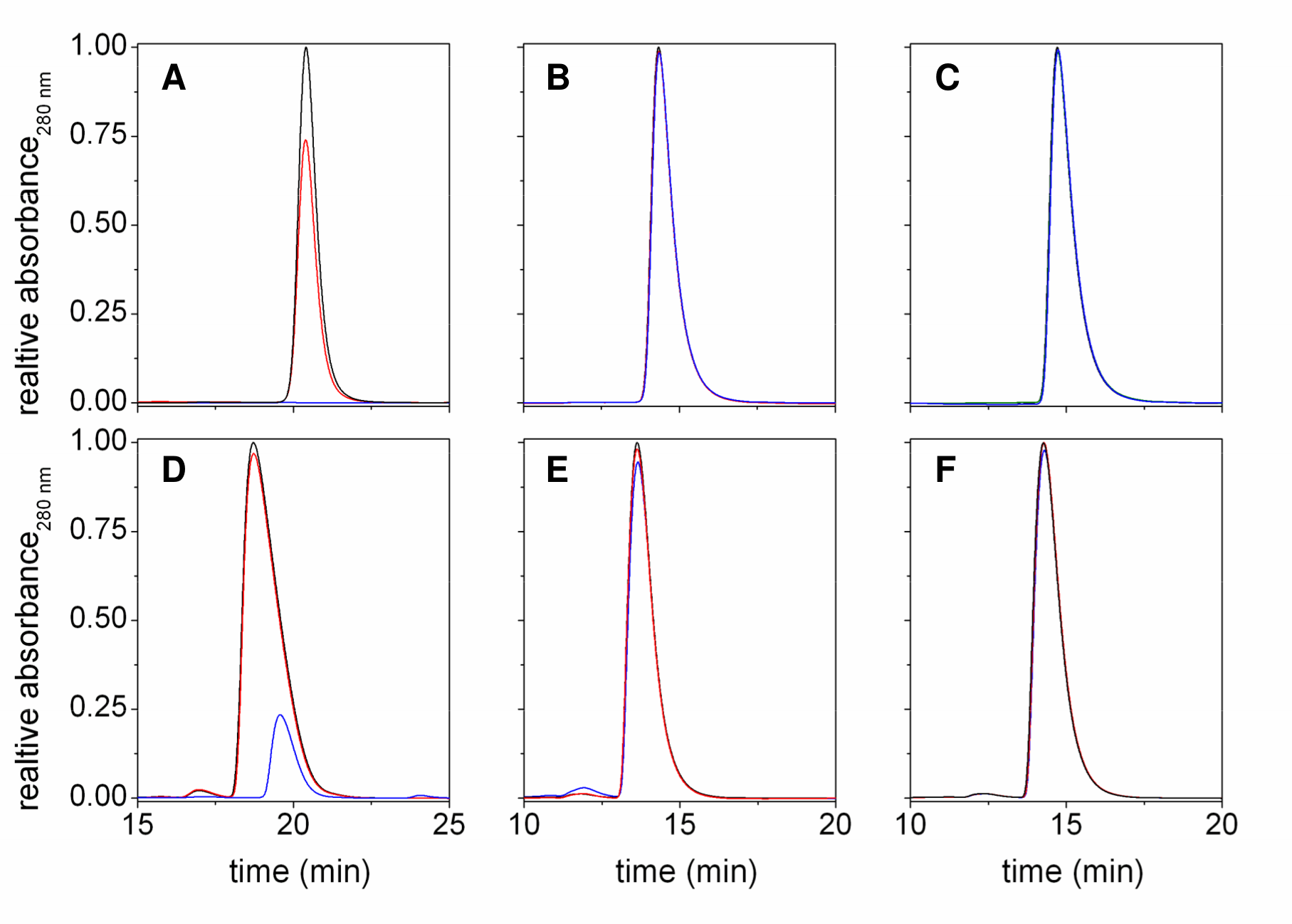

Supplement: Figure S5 — Overlay of SEC chromatograms of unstressed protein (black), stress level 1 (red) and stress level 2 (blue) material. Chromatograms are shown for (A) mAb1 HS, (B) mAb1 FT, (C) mAb1 S, (D) mAb2 HS, (E) mAb2 FT and (F) mAb2 S. HS: aggregates generated by heat and shake stress, FT: aggregates generated by freeze and thaw stress, S: aggregates generated by shear stress, mAb1: monoclonal antibody 1, mAb2: monoclonal antibody 2. (TIF) [file pone.0086322.s005.tif]

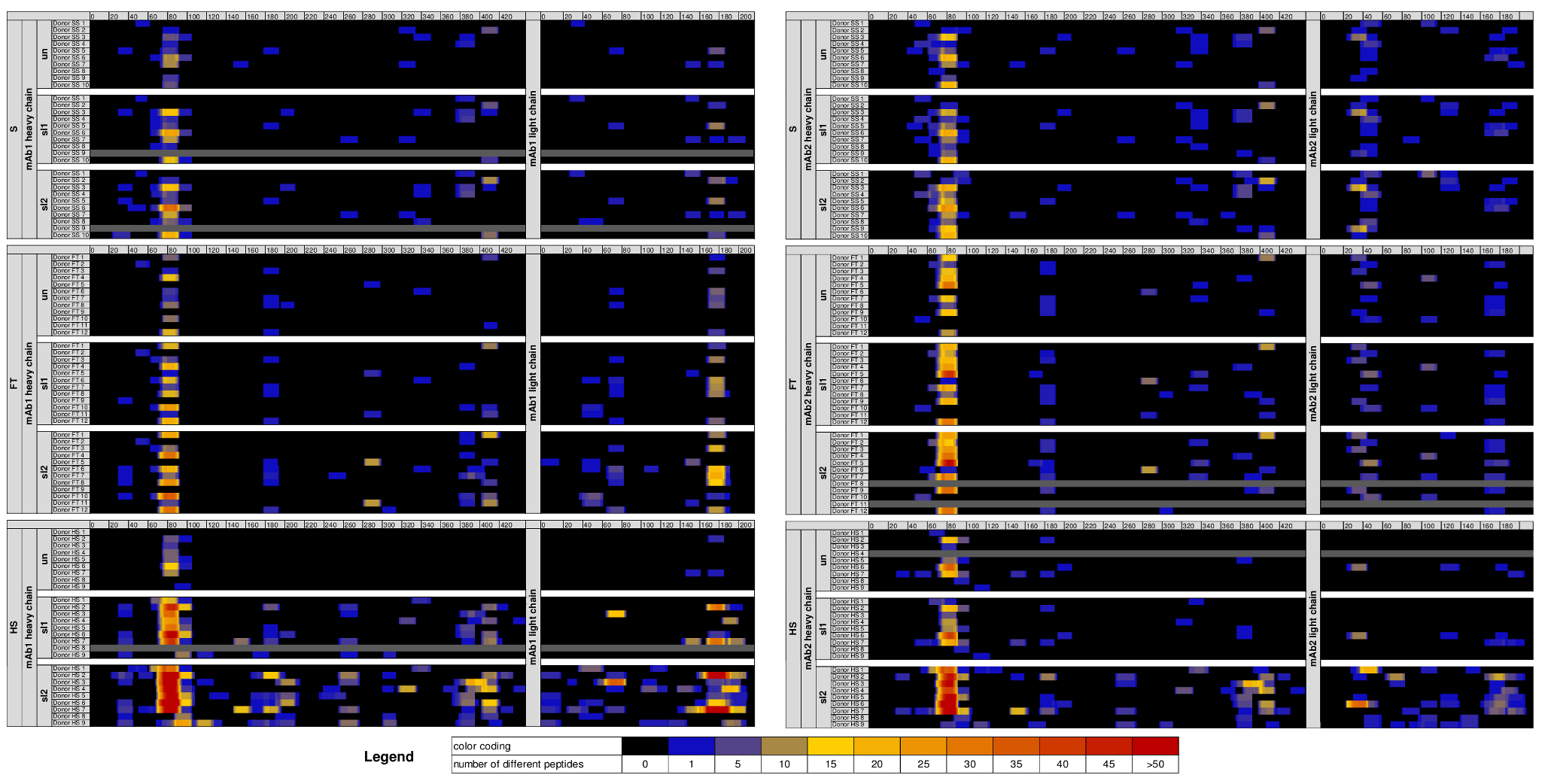

Supplement: Figure S6 — MAPPs heat map of identified HLA-DR associated peptides. Heat map visualization of mAb-derived HLA-DR associated peptides for both model antibodies. Each sequence position is colored according to the presence and number of different mAb-derived peptides identified. HS: aggregates generated by heat and shake stress, FT: aggregates generated by freeze and thaw stress, S: aggregates generated by shear stress, mAb1: monoclonal antibody 1, mAb2: monoclonal antibody 2, un: unstressed, sl1: stress level 1, sl2: stress level 2. (TIF) [file pone.0086322.s006.tif]
