# Supplementary material for: Aggregation of Human Recombinant Monoclonal Antibodies Influences the Capacity of Dendritic Cells to Stimulate Adaptive T-Cell Responses In Vitro
Source: PLoS One. 2014 Jan 21;9(1):e86322. doi: 10.1371/journal.pone.0086322 (PMC3897673; doi:10.1371/journal.pone.0086322)
Supplement: Table S1 — HLA DRB1 haplotypes of donors tested in MAPPs assay. (DOCX) [file pone.0086322.s007.docx]

Supplementary Table 1. HLA DRB1 haplotypes of donors tested in MAPPs assay.
